# Reinforcement Learning Model With Dynamic State Space Tested on Target Search Tasks for Monkeys: Self-Determination of Previous States Based on Experience Saturation and Decision Uniqueness

**DOI:** 10.3389/fncom.2021.784592

**Published:** 2022-02-04

**Authors:** Tokio Katakura, Mikihiro Yoshida, Haruki Hisano, Hajime Mushiake, Kazuhiro Sakamoto

**Affiliations:** ^1^Department of Physiology, Tohoku University School of Medicine, Sendai, Japan; ^2^Department of Neuroscience, Faculty of Medicine, Tohoku Medical and Pharmaceutical University, Sendai, Japan

**Keywords:** reinforcement learning, dynamic state space, target search task, experience saturation, decision uniqueness, exploration-exploitation trade-off, indefinite environment

## Abstract

The real world is essentially an indefinite environment in which the probability space, i. e., what can happen, cannot be specified in advance. Conventional reinforcement learning models that learn under uncertain conditions are given the state space as prior knowledge. Here, we developed a reinforcement learning model with a dynamic state space and tested it on a two-target search task previously used for monkeys. In the task, two out of four neighboring spots were alternately correct, and the valid pair was switched after consecutive correct trials in the exploitation phase. The agent was required to find a new pair during the exploration phase, but it could not obtain the maximum reward by referring only to the single previous one trial; it needed to select an action based on the two previous trials. To adapt to this task structure without prior knowledge, the model expanded its state space so that it referred to more than one trial as the previous state, based on two explicit criteria for appropriateness of state expansion: experience saturation and decision uniqueness of action selection. The model not only performed comparably to the ideal model given prior knowledge of the task structure, but also performed well on a task that was not envisioned when the models were developed. Moreover, it learned how to search rationally without falling into the exploration–exploitation trade-off. For constructing a learning model that can adapt to an indefinite environment, the method of expanding the state space based on experience saturation and decision uniqueness of action selection used by our model is promising.

## Introduction

Uncertainty is classified into two types. The first is where the state or probability space of the situation or environment is defined and fixed, as in the case of rolling a die. We cannot predict which roll will emerge, but we do know that a number from 1 to 6 will appear; thus, it is possible to utilize this prior knowledge. The other is the case where even the probability or state space of the environment is neither given nor hypothesized in advance. An environment with the latter type of uncertainty is defined as an indefinite environment, and adaptation to such an ever-changing indefinite environment is a critical issue for living systems (Shimizu, 1993).

**Figure 1:**
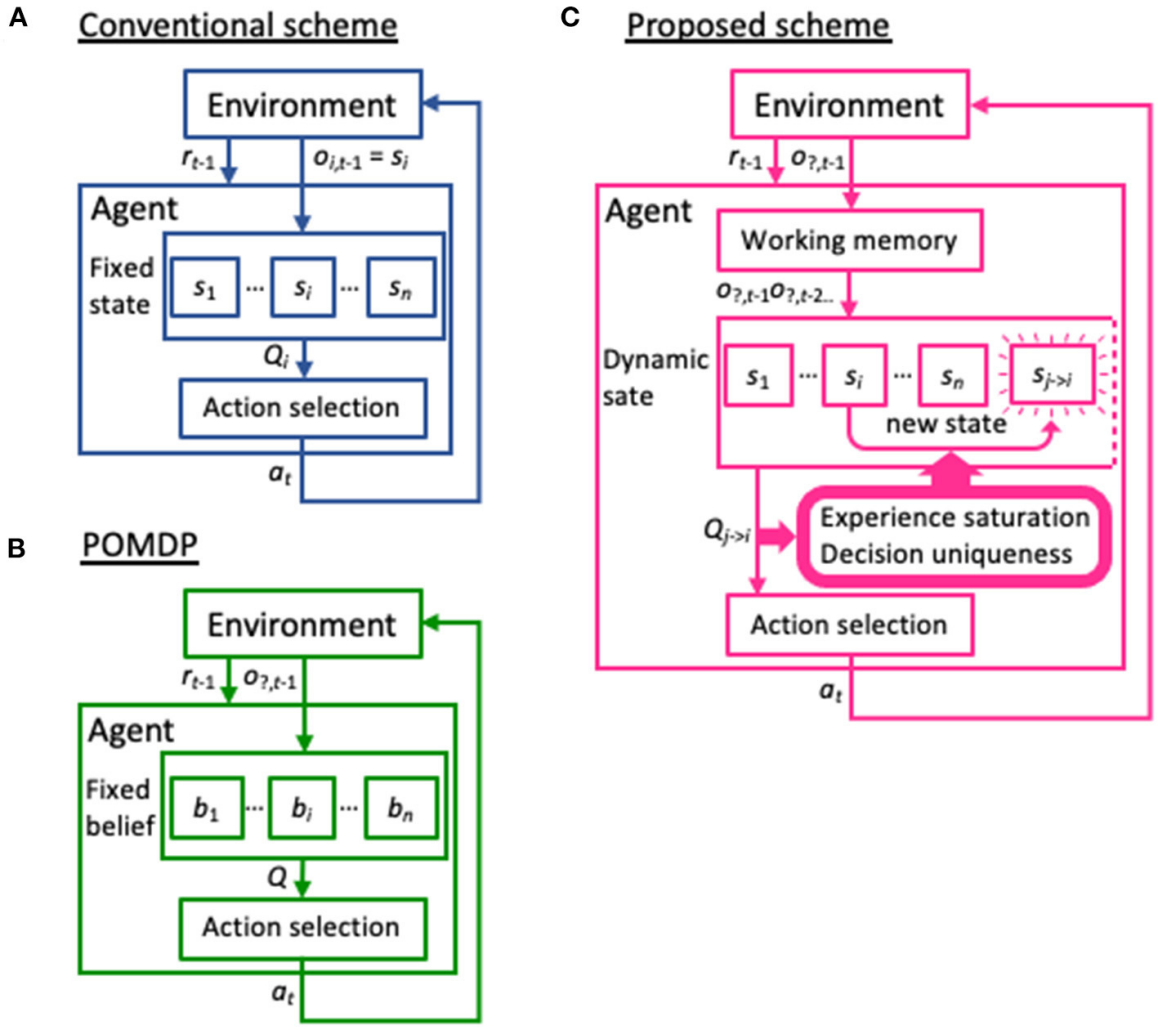
Differences in the basic schemes between previous models and the model presented in the current study. **(A)** In the conventional reinforcement learning scheme, observed variables and state variables are not distinguished. That is, the current state *s*_*i*_ is set based on the observation of the environment *o*__*i*_, t−1_ at the time *t*−1 and has a corresponding *Q*-table *Q*_*i*_, which provides an action *a*_*t*_. **(B)** In the partially observable Markov decision process (POMDP) model, the environment provides only a partial observation *o*_?, t−1_ to identify the current state. The agent has a set of beliefs or stochastic distribution for the possible states {*b*_*i*_}, and renews them through actions *a* and their reward outcomes *r*. Note that similar to the conventional scheme, the possible states are provided to the POMDP model in advance, in the form of beliefs. **(C)** Our dynamic state scheme also hypothesizes that the agent receives only partial information from the environment. However, unlike POMDP, these observations are temporarily stored in working memory and serve to generate a new state not prepared a priori, based on the two criteria experience saturation and decision uniqueness.

Learning is a primary ability of animals, allowing them to adapt to their environment. Reinforcement learning is a form of learning in which the agent learns to take a certain action in an uncertain environment, or without being explicitly informed of the correct answer. Instead, the agent learns a policy based on the state at the previous time-step to maximize the cumulative reward (Sutton and Barto, 1998). In particular, reinforcement learning models employing partially observable Markov decision process (POMDP) methods represent the most popular approach to coping with situations in which the current state is uncertain (Jaakkola et al., 1995; Thrun et al., 2005) and remain a thriving research area that attracts many researchers (e.g., Ahmadi et al., 2020; Bhattacharya et al., 2020; Bouton et al., 2020; Xie et al., 2020; Maliah and Shani, 2021). In particular, some recent POMDP models can learn policies from multiple past states or generate an infinite number of distributions within the probability or feature space, which has greatly improved the adaptability of machine learning to complex environments (Doshi-Velez, 2009; Doshi-Velez et al., 2015; Hausknecht and Stone, 2015; Azizzadenesheli et al., 2016; Igl et al., 2018). However, the current state of the environment functions as prior knowledge in reinforcement learning models. Even in the abovementioned advanced POMDP models, possible environmental states are generated within a given probability or feature space (Figures 1A,B). Therefore, these architectures may not achieve high learning performance in any unknown environment.

Infinite hidden Markov models (iHMMs) enable learning based on the arbitrary length of previous states without prior knowledge, even about the probability space (Beal et al., 2002; Teh et al., 2006; Mochihashi and Sumita, 2007; Mochihashi et al., 2009; Pfau et al., 2010; Doshi-Velez et al., 2015). This approach, by using Dirichlet process hierarchically, can dynamically generate states referring to arbitrary length of previous states, and is applied to, for example, inference of words within sentences (Mochihashi and Sumita, 2007; Mochihashi et al., 2009). However, this approach does not include explicit criteria for determining the appropriateness of state generation and its termination, raising concerns about whether it learns both dynamically and reproducibly.

With respect to our own experience, the state space is not uniquely and unilaterally provided to us in an indefinite environment; instead it is subjectively determined through our interaction with that environment. And even under such circumstances, we aim to ensure that the desired results are achieved. When we cannot obtain our desired outcomes, we will not blindly roll a die; instead, we seek an causal attribution (Heider, 1958; Kelley, 1967, 1973). That is, we will attempt to change our view of the situation we face or the state of the environment and make a deterministic or unique decision based on sufficient experience. This view is supported by findings in humans and animals: children prefer deterministic decisions, that is, infer unobserved causes whenever observed causes appear to act only stochastically (Schulz and Sommerville, 2006); when a rodent is presented an ambiguous conditional stimulus (CS) that is followed by an unconditional stimulus (US) in one context but not in another, the contextual information is recruited by the animal to determine the situation uniquely (Fanselow, 1990).

**Figure 2:**
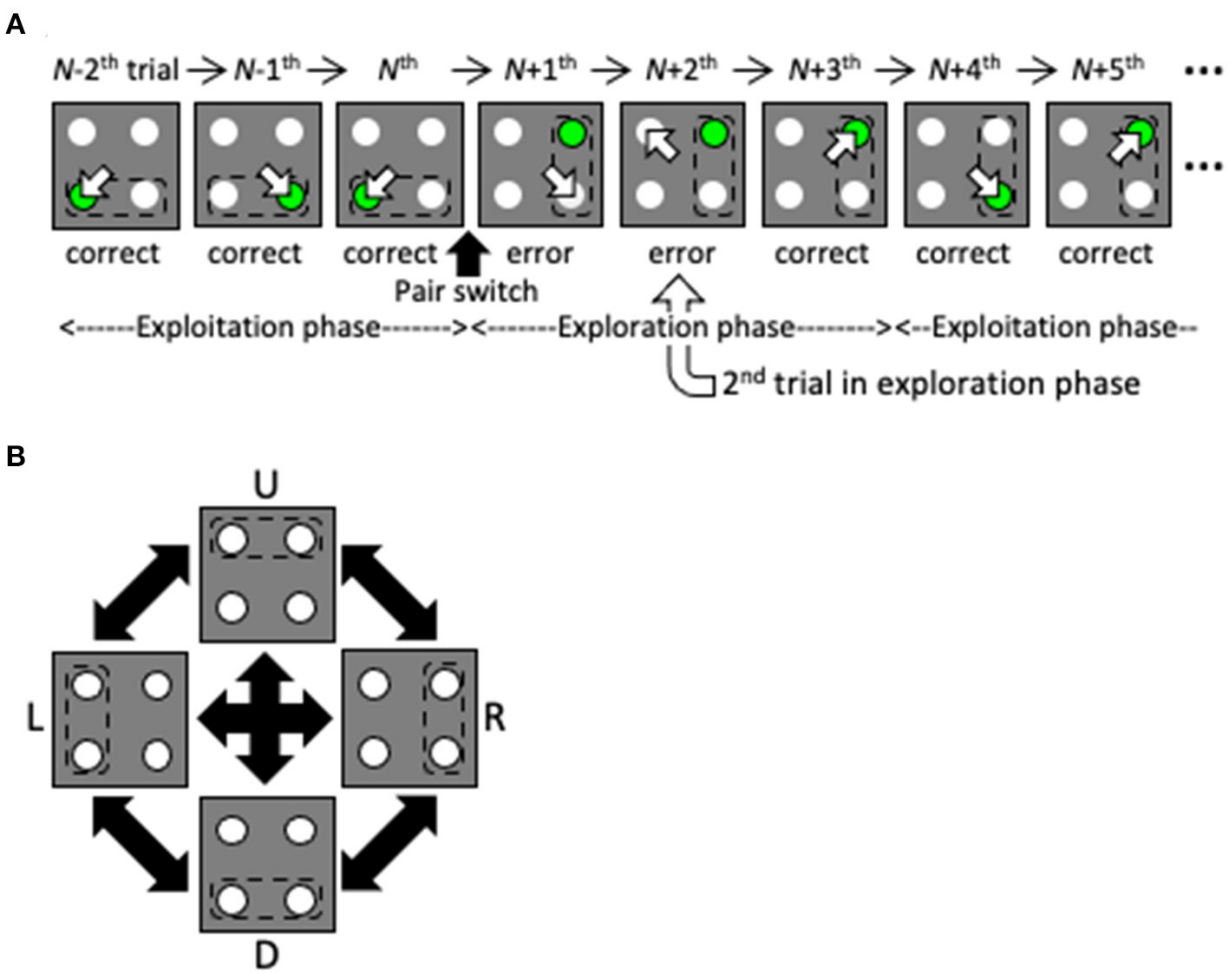
Overview of the two-target search task. **(A)** Schematic of several trials before and after a valid pair change. The pair change triggers the transition from the exploitation phase to the exploration phase. Dashed lines, empty arrows and green spots denote valid pairs, gazes and correct targets, respectively. Note that the subjects were not instructed to move their eyes by the green spot before gaze shift. **(B)** Valid pairs are randomly altered after a series of correct trials.

Here, we propose a reinforcement learning model with a dynamic state space that performs well in a two-target search task that was previously used in a physiological experiment with non-human primates (Kawaguchi et al., 2013, 2015; Figure 2, see also Supplementary Figure 1). Briefly, subjects were required to gaze at one light spot from among four identical stimuli. If the correct spot (designated by green in Figure 1) was selected, a reward was delivered. After training, the subjects learned to saccade alternately to two targets in a valid pair, and received a reward for several correct trials in a row (the exploitation phase). If the valid pair was changed without instruction, they started searching for a new valid pair after making errors (the exploration phase). In this task, by simply hypothesizing that the previous state is the previous trial, the agent cannot maximize the total reward. To do so, the agent must consider the two previous trials together as the previous state. Thus, this task can address the issue of how the two previous trials together can come to be regarded as the previous state without prior knowledge of the task structure. Our proposed model is given no prior knowledge of the task structure other than the action of gazing at one of the four spots. Instead, it starts learning using the immediately preceding trial as the starting state, and expands and contracts the state space in the direction of previous trials based on the criteria of experience saturation and the decision uniqueness of the action selection (Figure 1C). The model performed comparably to the optimal model, in which prior knowledge of the task structure was available. We consider the dynamic learning mechanism proposed in this study to be a crucial component for systems to adapt to indefinite environments.

## Methods

### The Reinforcement Learning Model

We developed a reinforcement learning model with a dynamic state space. The basic structure of the model was grounded in the conventional reinforcement learning (Rescorla and Solomon, 1967) as follows. The action value function, *Q*(*S*_*N*_ = *s*_*i*_, *A*_*N*_ = *a*_*j*_) for the pair of a particular state, *s*_*i*_, and an action, *a*_*j*_, at the *N*th trial were updated by the following equation:


(1) $$\begin{array}{l} Q\left(S_{N+1},A_{N+1}\right)\leftarrow Q\left(S_{N},A_{N}\right)+\alpha \delta \left(S_{N},A_{N}\right), \end{array}$$

where α is the learning rate, set to 0.1 in the range that showed desirable results revealed by the parameter search (see **Figure 7**). δ is the reward prediction error, given by


(2) $$\begin{array}{l} \delta \left(S_{N},A_{N}\right)\equiv r-Q\left(S_{N},A_{N}\right), \end{array}$$

where *r* is the reward delivered for *A*_*N*_ taken at *S*_*N*_ in the *N*th trial. If the correct spot was selected, a reward *r* = 1 was delivered, otherwise *r* = 0 was given. In the following, we will refer to whether a reward has been obtained or not as (reward) outcome. *A*_*N*_ was selected according to the stochastic function, *P*^π^(*A*_*N*_ = *a*_*j*_|*S*_*N*_ = *s*_*i*_), under *S*_*N*_ = *s*_*i*_. A policy, π, i.e., *P*^π^ is the softmax function, defined by


(3) $$\begin{array}{l} P^{\pi }\left(a_{j}|s_{i}\right)\equiv \frac{\exp\left(\beta Q\left(s_{i},{\;a}_{j}\right)\right)}{\sum_{k}^{4}\exp\left(\beta Q\left(s_{i},{\;a}_{k}\right)\right)}, \end{array}$$

where the parameter β, termed the inverse-temperature, was set to 7 in the range that provided desirable results revealed by the parameter search (see **Figure 8**). For action selection, the state that refers to the longest history of recent trials among generated states was used.

### Expanding and Contacting the State Space

**Figure 3:**
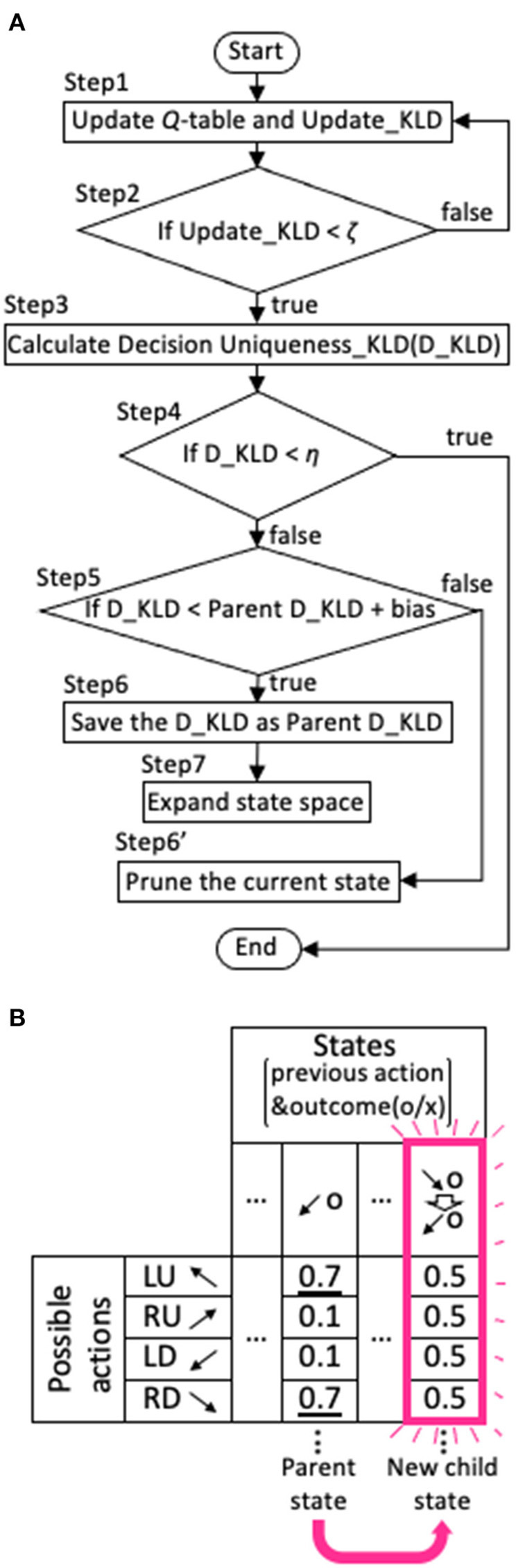
Expansion and contraction of the state space. **(A)** Flowchart of the expansion and contraction process. **(B)** An example of state expansion derived from a parent state in *Q*-table. The direction of the arrow represents the target that the agent looked at, and o and x represent the correct answer and error, respectively. The example in the figure shows that a new state is generated from the state that the agent looked at LD and was rewarded one trial ago, to the state that it looked at LD and was rewarded one trial ago after it looked at RD and was rewarded two trials ago. The numbers in the *Q*-table represent *Q*-values. The initial *Q*-value for each action is set to 0.5.

Our model was designed to avoid the need for stochastic decisions as much as possible. Specifically, when the model did not have a value function for a particular action that required a much larger value compared with others following extensive experience with the state, it expanded the range of the state backward in time. We illustrate the algorithm of this expansion in Figure 3A.

The initial state space of the model calculation was set as a particular combination of the four possible actions, namely gazing at the right-up (RU), left-up (LU), left-down (LD), or right-down (RD) spot, and the outcome (correct or error) from one trial. The initial *Q*-value for each action was set to 0.5. The model monitored the stochastic mean policy for each state *s*_*i*_, given by


(4) $$\begin{array}{l} P_{mean,N_{update,s_{i}}}^{\pi }\left(\text{a}|s_{i}\right)\equiv \frac{1}{N_{update,s_{i}}}\overset{N_{update,s_{i}}}{\underset{l=1}{\sum }}P_{l}^{\pi }\left(\text{a}|s_{i}\right) \end{array}$$

where *N*_*update,si*_ is the number of times that the *Q*-values for the state *s*_*i*_ were updated. Then, it calculated the information gain or the Kullback-Leibler divergence (KLD) obtained by updating the stochastic policy (step 1 in Figure 3A):


(5) $$\begin{array}{l} {Update\text{\_}KLD}_{s_{i}}\\ \left(P_{mean,N_{update,s_{i}}}^{\pi }\left(\text{a}|s_{i}\right)|{|P}_{mean,N_{update,s_{i}}-1}^{\pi }\left(\text{a}|s_{i}\right)\right)\\ \equiv \;\overset{4}{\underset{j}{\sum }}P_{mean,N_{update,s_{i}}}^{\pi }\left(a_{j}|s_{i}\right)\log\frac{P_{mean,N_{update,s_{i}}}^{\pi }\left(a_{j}|s_{i}\right)}{P_{mean,N_{update,s_{i}}-1}^{\pi }\left(a_{j}|s_{i}\right)}. \end{array}$$

We referred to this as the Update_KLD. *N*_*update,si*_ - 1 indicates the number of trials since the model last encountered state *s*_*i*_ and calculated the mean *P*(***a***|*s*_*i*_). We used the Cesàro average to test whether the decision uniqueness would be improved by promoting state expansion when the number of trials experienced in the state of interest becomes large.

Next, the model judged whether the Update_KLD of the state *s*_*i*_ fell below the criterion for experience saturation, ζ (step 2),


(6) $$\begin{array}{l} {Update\text{\_}KLD}_{s_{i}}<\;\zeta \end{array}$$

indicating that information can no longer be gained by updating. The value of ζ was determined to be 10^−6^ in the range that showed desirable results revealed by the parameter search (see **Figure 5**). When the Update_KLD_*si*_ was < ζ, the distribution of $P_{mean,N_{update,s_{i}}}^{\pi }\left(\text{a}|s_{i}\right)$ was compared with $P_{ideal}^{\pi }\left(\text{a}|s\right)$. $P_{ideal}^{\pi }\left(\text{a}|s\right)$ is the action selection probability that only one action will be selected and was obtained as follows. First, the ideal policy, *Q*_*ideal*_(***a***|*s*_*i*_), was obtained by setting the largest value within *Q*(***a***|*s*_*i*_) to 1 and the other values to zero. For example, if the *Q*(***a***|*s*_*i*_) were, {0.1, 0.4, 0.2, 0.1}, the *Q*_*ideal*_(***a***|*s*_*i*_), would be set to {0, 1, 0, 0}. Thereafter, the $P_{ideal}^{\pi }\left(\text{a}|s\right)$ was calculated from *Q*_*ideal*_(***a***|*s*_*i*_) using the softmax function in Equation (3). For comparison, another KLD was calculated, as described below (step 3):


(7) $$\begin{array}{l} {D\text{\_}KLD}_{s_{i}}\left(P_{mean,N_{update,s_{i}}}^{\pi }\left(\text{a}|s_{i}\right)|{|P}_{ideal}^{\pi }\left(\text{a}|s\right)\right)\\ \equiv \underset{j}{\sum }P_{mean,N_{update,s}}^{\pi }\left(a_{j}|s_{i}\right)\log\frac{P_{mean,N_{update,s}}^{\pi }\left(a_{j}|s_{i}\right)}{P_{ideal}^{\pi }\left(a_{j}|s\right)} \end{array}$$

We called this the Decision-uniqueness KLD (D_KLD). When the D_KLD was below the criterion for a preference for deterministic action selection, η (step 4),


(8) $$\begin{array}{l} {D\text{\_}KLD}_{s_{i}}<\;\eta \end{array}$$

the agent had uniquely selected an action for state *s*_*i*_, and the *Q*-table was not expanded any further. η was set to 3, the median of the range between values of > 1 and <5, which produced fair performance revealed by the parameter search (see **Figure 6**). When the D_KLD did not meet the criterion, it was also compared to the parent D_KLD (step 5), defined as the D_KLD of the parent state from which the current state *s*_*i*_ had been expanded (e.g., Figure 3B). In step 6, when the D_KLD is judged to be less than its corresponding Parent D_KLD, as in Equation (9),


(9) $$\begin{array}{l} {D\text{\_}KLD}_{s_{i}}<\;{Parent\;D\text{\_}KLD}_{s_{i}}+bias \end{array}$$

the D_KLD value is saved as the parent D_KLD, and the state is expanded as depicted in the *Q*-table of Figure 3B (step 7). That is, the new state (child state) is the combination of the parent state and the state of one more previous trial to which the parent state refers. In the schematic example in Figure 3B, a new state is generated from the state that the agent looked at LD and was rewarded one trial ago, to the state that it looked at LD and was rewarded one trial ago after it looked at RD and was rewarded two trials ago. The initial *Q*-value for each action is set to 0.5. On the other hand, if Equation (9) does not hold, the current state being processed (see flowchart in Figure 3A) is pruned (step 6'). When the current state consists of only the previous one trial, it is not erased because there is no parent state with which it could be compared. The bias is set to be −1 in all calculation except in the case shown in **Figure 9**.

### Models Used for Comparison

In the Results section, we compare our dynamic state model with three models with state spaces of fixed sizes. The first model was called the fixed 4-state model, the state space of which consisted of four elements. In other words, this model selected the next action based on the previous four possible actions, ignoring their reward outcomes. We called the second model the fixed 8-state model, which had a state space comprising eight elements, that is, the combination of four actions and their outcomes (i.e., correct or error) from the previous trial. In any case, since the action selection in these two models was based only on the previous one trial, they did not show good performance in the two-target search task. By contrast, as the best model for the two-target search task (because it assumed that the task structure was known and made decisions based on the actions and reward outcomes of the last two trials), the “fixed 8by8-state model” was used to evaluate the performance of our dynamic state model.

For further comparison, we also created a simple POMDP model (Thrun et al., 2005). The model referred only to the action of the previous one trial and its reward outcome (eight total cases). Instead, to estimate the current valid pair, the belief for each target pair (i.e., right [R], left [L], up [U], and down [D] pairs; Figure 2B) was calculated. Specifically, the value of the belief, *b*_*k*_, about the pair *k* inferred from the previous gaze (e.g., the R pair and U pair in the case of RU) was increased or decreased depending on the reward outcome, while normalizing the total of the beliefs to 1. Because the above-mentioned eight cases existed for each of the four possible pairs, the *Q-*table consisted of 32 total rows. Then, the composite *Q*-value was obtained as


(10) $$\begin{array}{l} \overset{4}{\underset{k}{\sum }}b_{k}Q_{k}\left(s_{i},a_{j}\right) \end{array}$$

the sum of the 4 rows within the *Q*-table corresponding to the previous state *s*_*i*_, weighted by the relevant belief *b*_*k*_. The next action was selected by substituting the composite *Q*-value into the softmax function (3). The *Q*-value for the selected action *a*_*j*_ and each belief *b*_*k*_ was updated using the reward prediction error δ_*k*_, as in Equation (2), multiplied by the learning rate and belief, α*b*_*k*_δ_*k*_(*s*_*i*_, *a*_*j*_ ).

We also compared the proposed model with iHMMs, which hierarchically use a Dirichlet process as models to dynamically generate states based on history without prior knowledge (Supplementary Figure 2A). The model starts from the base state, which has no defining conditions, and probabilistically generates new states (Supplementary Figure 2B). As in the proposed model, states other than the base state consist of combinations of previous actions and their reward outcomes. Nodes consisting of an action and its result of one previous trial are generated directly below the base state. Below these nodes are nodes that refer to the information of the last two trials. As the tree further branches downward, the number of trials to be referred to increases. The Chinese restaurant process was used to implement the Dirichlet process (Supplementary Figures 2C,D) (Teh et al., 2006). Tables (filled tables in Supplementary Figures 2C,D) were prepared for each of the four possible actions, at each of which guests were seated (represented by the people with filled heads in Supplementary Figures 2C,D). If the executed action is correct, a new guest will be seated at the table. In a state *s*_*i*_, each action *a*_*j*_ is selected with the following probability, where *g*_j_ is the number of guests seated at the corresponding table:


(11) $$\begin{array}{l} P\left(a_{j}|s_{i}\right)=\frac{g_{j}}{\sum_{k}^{4}g_{k}+\lambda } \end{array}$$

The new state (child state), which considers one more previous trial, is generated from the current state (parent state) with the following probability:


(12) $$\begin{array}{l} \frac{\lambda }{\sum_{k}^{4}g_{k}+\lambda } \end{array}$$

where λ is the concentration parameter. In the Dirichlet process version (Supplementary Figure 2C), the initial child state has one unique guest (presented by a person with a brank head in Supplementary Figure 2C) in each action table, i.e., there is a uniform distribution. On the other hand, in the hierarchical Dirichlet process (Supplementary Figure 2D), the initial value of the child state is given by the distribution of the parent state. The latter is more popular, however, the former method was also used for a fairness of comparison, because in the proposed model, the child state does not inherit the *Q*-value of the parent state for evaluating the amount of learning (see **Figure 13**). In contrast to the proposed model, these two iHMMs are vulnerable to the parameter λ. After preliminary calculations, we selected 0.2 as the λ value, where the Dirichlet process version performed closest to the proposed model (see **Figure 12**). Similar to the proposed model, the end of the tree, i.e., the state referring to a large number of trials, was preferentially used for action selection. If the selected action was incorrect, the guest was removed from the table, and the state including a guestless table was pruned. However, state pruning was not observed in the simulation.

### Supplementary Explanation of the Two-Target Search Task

In the two-target search task that the animals actually performed, each trial consisted of a sequence of events of 500 ms in duration, including a fixation period. However, for simplicity, in the present study, one time step of calculation was set to one trial (i.e., a combination of events in which the agent takes an action and obtains its reward outcome). When an incorrect spot was chosen, the same trial was repeated until the correct target was found. The valid pair was switched pseudo-randomly. The number of consecutive correct trials required before the valid-pair switch was set to seven, as in our previous physiological experiments involving monkeys.

## Results

### Performance of the Proposed Model

**Figure 4:**
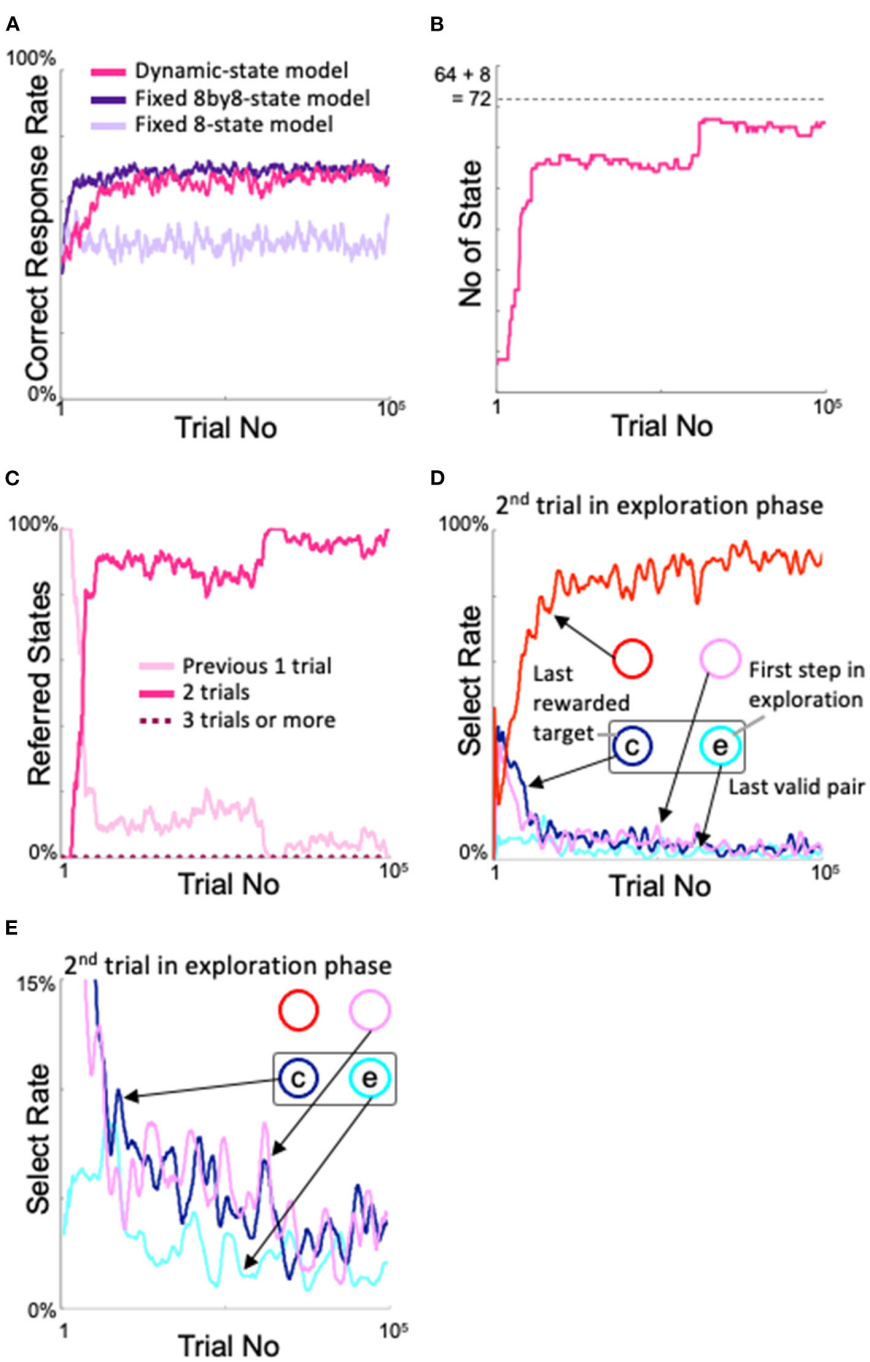
Changes in the proposed model with learning. **(A)** Time course of the correct response rate and comparison with fixed state models. **(B)** Increase in the number of states. **(C)** Changes in the states referred to in each action selection. **(D)** Analysis of the model's behavior during the second trial of the exploration phase. “c” and “e” denote correct and error response, respectively. **(E)** Enlarged view of the 0–15% area of the select rate in **(D)**.

In Figure 4A, we show the changes in percentage of correct responses from the start of learning of the models. The fixed 8by8-state model is the ideal learner for the two-target search task, i.e., with 8×8 = 64 states, so it quickly learned the current pair and obtained a high correct response rate, with the upper limit close to the theoretical value. On the other hand, the fixed 8-state model has only eight states, which are the combination of the gaze action and its reward outcome on the previous trial. In the two-target search task, even if the correct answer is obtained by looking at one target, two targets might be correct in the next trial. In this sense, the fixed 8-state model is not an ideal learner for the two-target search task. In fact, the correct response rate was not as good as the fixed 8by8-state model. Our proposed dynamic state model exhibited a slower increase in the correct response rate than the fixed 8by8-state model, but its performance was comparable to that of the ideal model after approximately 20,000 trials.

The state was expanded based on experience saturation and action decision uniqueness. The number of states of the dynamic state model showed a change corresponding to the change in the correct response rate (Figure 4B). At the very beginning of the learning process, the dynamic state model had the same eight states as the fixed 8-state model, but the number of states began to increase rapidly around the 3,000th trial; by the time the correct response rate was comparable to that of the fixed 8by8-state model, the number of states had almost stopped increasing. Microscopically, there were also many places where the number of states slightly decreased, which indicates that the model was appropriately pruning unnecessary states. The fact that the final number of states did not exceed 8 + 8^2^ = 72 (dashed line in Figure 4B), which means that the model refers to less than three trials, indicates that the model appropriately expands and contracts the number of states. Figure 4C shows the number of former trials that the model referenced for action selection. The fact that it did not refer to more than two trials indicates that the number of states is not expanded more than necessary.

The increase in performance with learning of the dynamic state model is thought to correspond to an increase in sophisticated searching for novel target pairs during the exploration phase. To examine this, we analyzed where the model looked during the second trial of the exploratory phase (see Figure 2A). Figure 4D shows that, in the second trial of the exploration phase, as the correct response rate increased, the model had a high probability of looking at the diagonal side of the incorrect gaze during the first trial of the exploration phase (“e” in Figures 4D,E); below, this will be referred to as the diagonal spot in the second trial of the exploration phase. This choice of diagonal gaze is reasonable; a valid pair always comprised two neighboring spots, so if the agent correctly answered two trials ago and incorrectly one trial ago, then the probability was high that the other neighboring spot of the correct target two trials ago (“c” in Figures 4D,E), i.e., the diagonal side of the spot that was incorrect one trial ago, was the correct answer. The behavior of this model was also in good agreement with the behavioral results from our monkey experiments (Figure 3 of Kawaguchi et al., 2015). This sophisticated behavior during the exploration phase means that the model did not stick rigidly to the most recent valid pair, nor did it suddenly “roll a die.” In other words, in the dynamic state model (and the ideal model, i.e., the fixed 8by8-state model), and in monkeys that perform the two-target search task, exploration and exploitation are not in a trade-off relationship; rather, the models and monkeys learned how to search.

Looking back at Figure 4B, we can see a step-like increase in the number of states after the 60,000th trial, although the correct rate did not considerably differ (Figure 4A). This corresponded to a decrease in access to the state that considers only the previous trial (Figure 4C). In more detail, during this period, access to the “correct answer two trials ago → incorrect answer one trial ago” state increased instead of the “incorrect answer one trial ago” state. This step-like increase was reflected in a slight increase in the diagonal gaze in the second trial in the exploration period (Figure 4D) due to further refinement of the exploration behavior. Figure 4E shows an enlargement of the lower part of Figure 4D. Notably, the probability of gazing at spots other than the diagonal spot decreased further in the second trial of the exploration period after approximately 60,000 trials, although variation was observed in each line.

### Determination of the Ranges of Meta-Parameters for Desirable Model Performance

Our dynamic state–space model showed good performance in a sufficiently wide range for each of the ζ, η, α, and β meta-parameters. Below, we will show how the model behaves beyond and below the default range.

**Figure 5:**
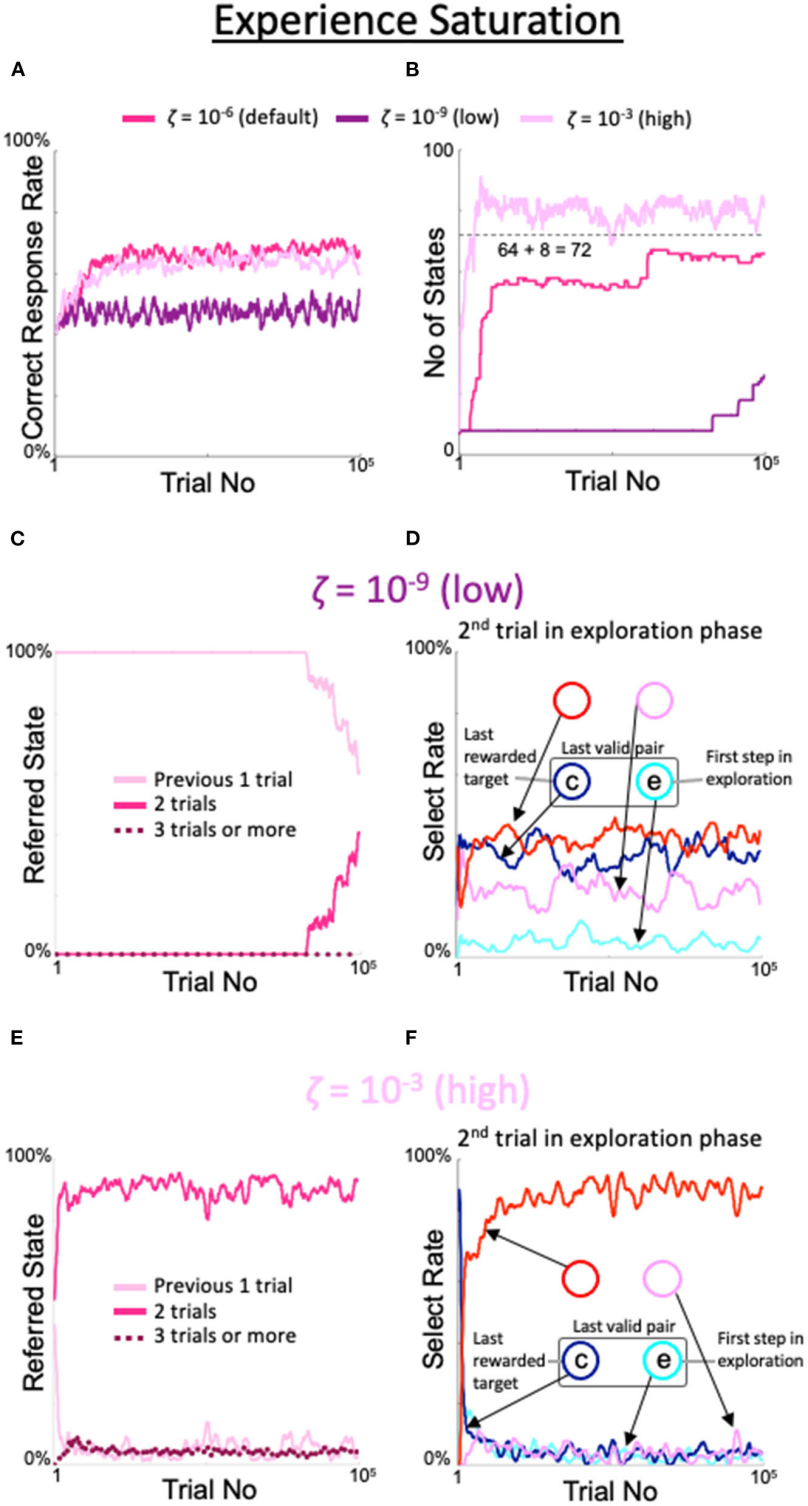
Effects of threshold modulation of experience saturation. Formats are identical to Figure 4. **(A)** Correct response rates. **(B)** Corresponding number of states. **(C,D)** States referred to in each action selection **(C)** and analysis of the model's behavior in the second trial of the exploration phase **(D)** at a low value of ζ = 10^−9^. **(E,F)** Same plots as **(C,D)** for a high value of ζ = 10^−3^.

When the criterion for experience saturation ζ was set lower than the default value of 10^−6^, the increase in the correct rate was delayed. When ζ was 10^−9^, the rate of correct answers was similar to the rate of correct answers for the fixed 8-state model (dark purple line in Figure 5A). Similarly, the number of states stayed at eight for an extended period and finally began to increase after 80,000 trials (dark purple line in Figure 5B). Notably, this corresponded to the persistence of the period in which the model referred only to the previous one trial (Figure 5C). In the second trial of the exploration period, the rate of gazing at the spot diagonally opposite the spot gazed at during the first trial was also low (red line in Figure 5D). In contrast, when ζ was set higher than the default value, the number of trials referenced exceeded two, while the correct rate did not substantially change. When ζ was set to 10^−3^, the rate of correct responses was only slightly lower than when ζ was set to the default value, and the gaze pattern in the second exploratory trial was similar to the default gaze pattern (Figure 5F). However, the number of states that increased faster than the default was limited but exceeded 8 + 8^2^ = 72 (light purple line in Figure 5B); indeed, the results of the last three trials were referenced with a small but distinct probability (dotted line in Figure 5E).

**Figure 6:**
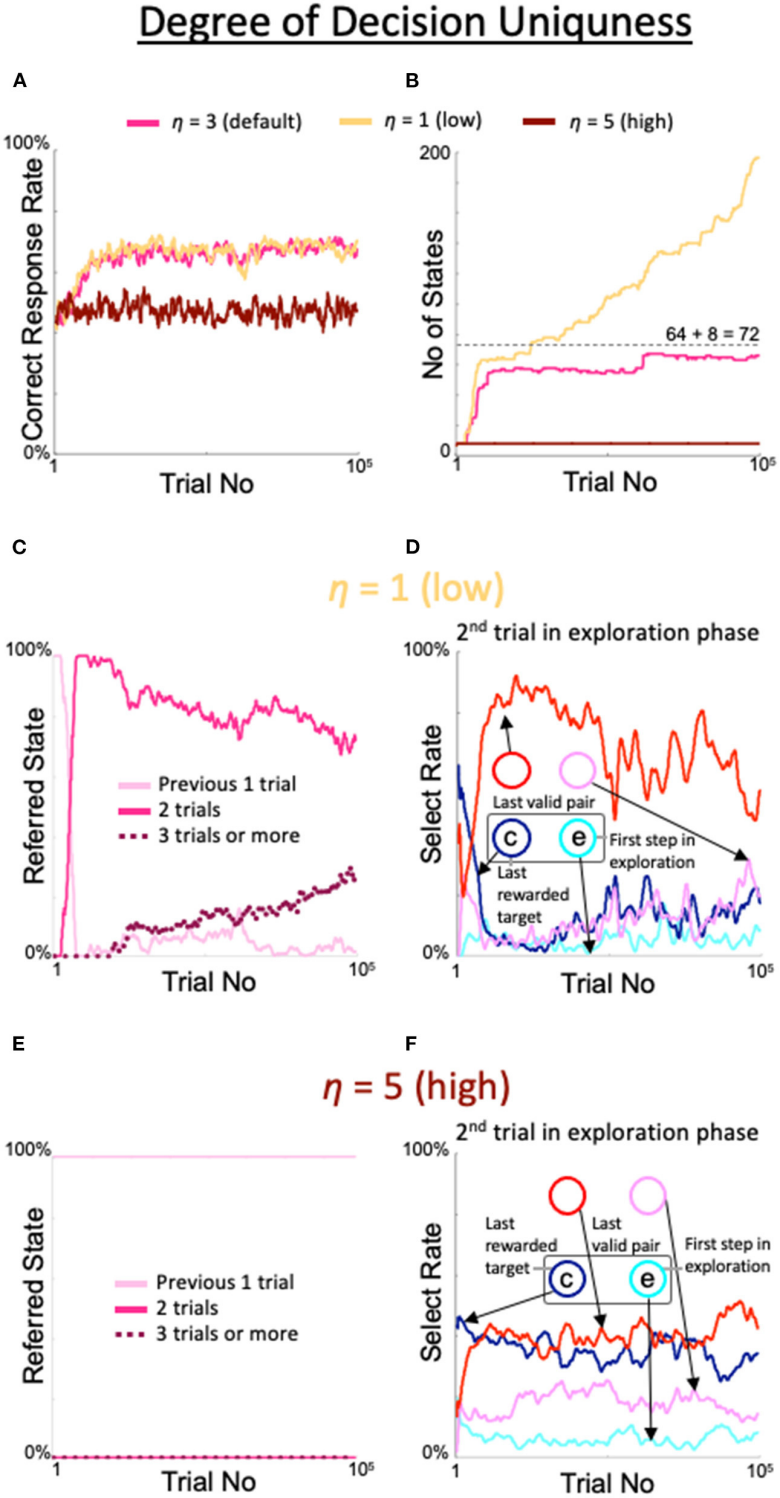
Effects of threshold modulation of the degree of decision uniqueness. Formats are identical to Figure 5. **(A)** Percentage of correct answers. **(B)** Changes in number of states. **(C,D)** States referred to in each action selection **(C)** and the model's behavior in the second trial of the exploration phase **(D)** at a low value of η = 1. **(E,F)** Same plots as **(C,D)** for a high value of η = 5.

The excessive expansion and de-expansion of the number of states observed above was also obtained by varying the threshold for the degree of decision uniqueness. When η was set to 1, which was smaller than the default value, the correct response rate was similar to the correct response rate with the default value (yellow line in Figure 6A); however, the number of states increased rapidly over the 100,000 trials (yellow line in Figure 6B) and the rate of referring to the results of the last three trials continued to increase (dotted line in Figure 6C). Associated with the increase in this rate, the rate of diagonal gaze in the second trial of the exploration phase deteriorated (red line in Figure 6D). In contrast, when η was set to 5, a larger value than the default, the percentage of correct responses (brown line in Figure 6A), number of states (brown line in Figure 6B), number of immediate trials referenced (pink line in Figure 6E), and behavior during the second trial of the exploration period (Figure 6F) were all identical to the findings in the fixed 8-state model.

**Figure 7:**
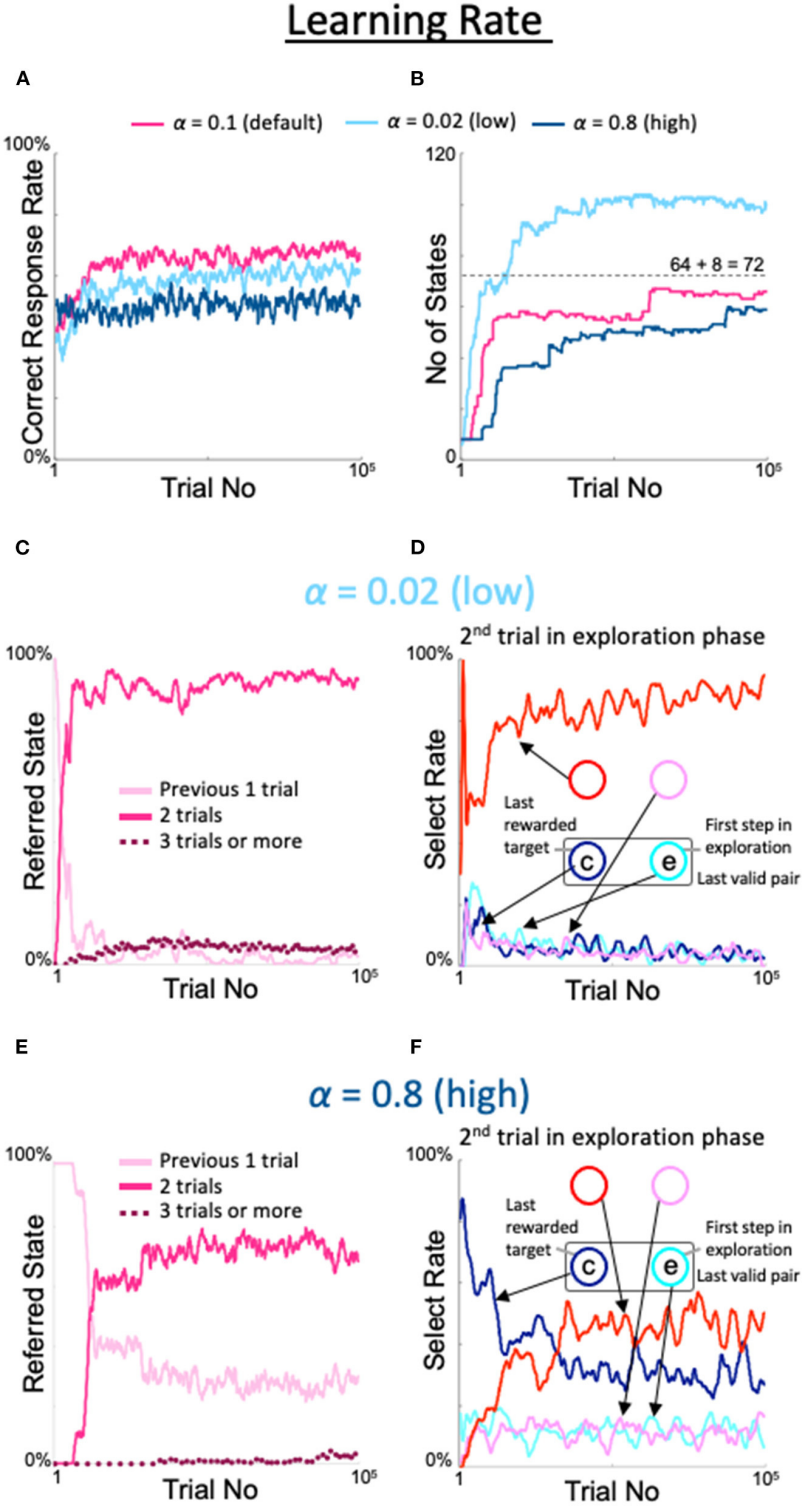
Effects of modulation of learning rate. Formats are identical to Figures 5, 6. **(A)** Percentage of correct answers. **(B)** Changes in number of states. **(C,D)** States referred to in each action selection **(C)** and the model's behavior in the second trial of the exploration phase **(D)** at a low value of α = 0.02. **(E,F)** Same plots as **(C,D)** for a high value of α = 0.8.

We also examined the effects of changing the learning rate α, a conventional meta-parameter for reinforcement learning. When α was set to the lowest possible value (0.02), the correct response rate was slightly lower than the rate observed with the default value (light blue line in Figure 7A), the number of states was limited but exceeded 8 + 8^2^ = 72 (light blue line in Figure 7B), the last three trials were referenced at a low but nearly constant rate (dotted line in Figure 7C), and the diagonal spot was gazed at frequently in the second trial of the exploration period (red line in Figure 7D). However, the highest value (α = 0.8) showed a peculiar property not described above. In particular, the correct response rate was higher than in the fixed 8-state model but lower than in the dynamic state–space model (dark blue line in Figure 7A). The number of states also increased, although it was lower than in the dynamic state–space model (dark blue line in Figure 7B). Importantly, the rate at which only the previous trial was referenced did not substantially decrease (pink line in Figure 7E), although the last three trials were rarely but sometimes referenced (dotted line in Figure 7E). Therefore, the diagonal gaze rate did not increase enough in the second trial of the exploration period (red line in Figure 7F).

**Figure 8:**
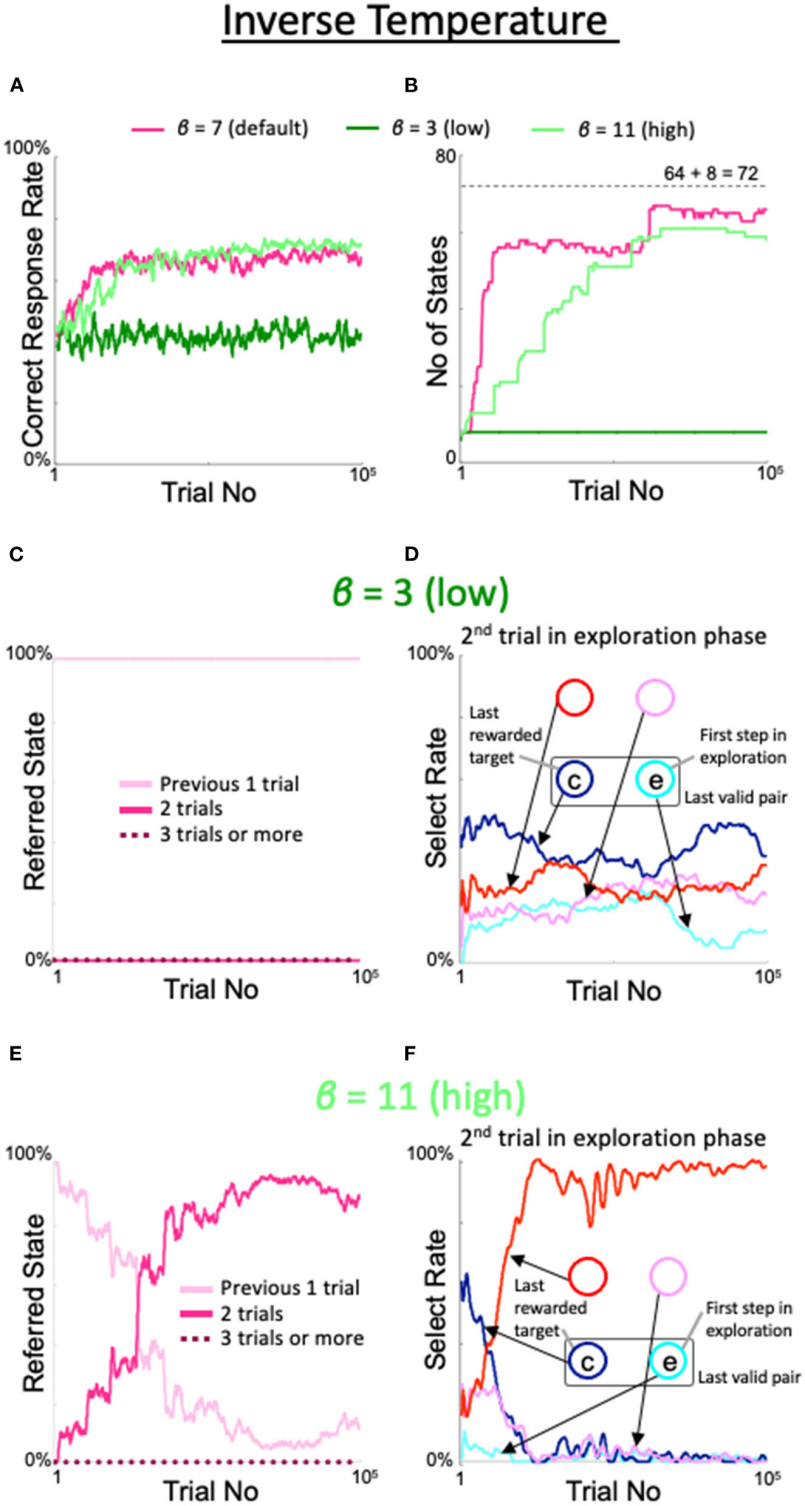
Effects of modulation of inverse temperature in the softmax function used for action selection. Formats are identical to Figures 5–7. **(A)** Percentage of correct answers. **(B)** Changes in number of states. **(C,D)** States referred to in each action selection **(C)** and the model's behavior in the second trial of the exploration phase **(D)** at a low value of β = 3. **(E,F)** Same plots as **(C,D)** for a high value of β = 11.

The effects of varying the inverse temperature β in the softmax function for action selection were as follows. When β was lowered from the default value to 3, the correct response rate (dark green line in Figure 8A), number of states (dark green line in Figure 8B), number of immediate trials referenced (pink line in Figure 8C), and the behavior during the second trial of the exploration period (Figure 8D) all exhibited the same properties as observed in the fixed 8-state model. However, when the inverse temperature was increased to 11, i.e., beyond the range of desirable results, the increases in the correct response rate (light green line in Figure 8A), number of states (light green line in Figure 8B), rate referencing the last two trials (red line in Figure 8E), and rate of diagonal gaze during the second trial of the exploration period (red line in Figure 8F) were not bad, but delayed compared with the default case.

**Figure 9:**
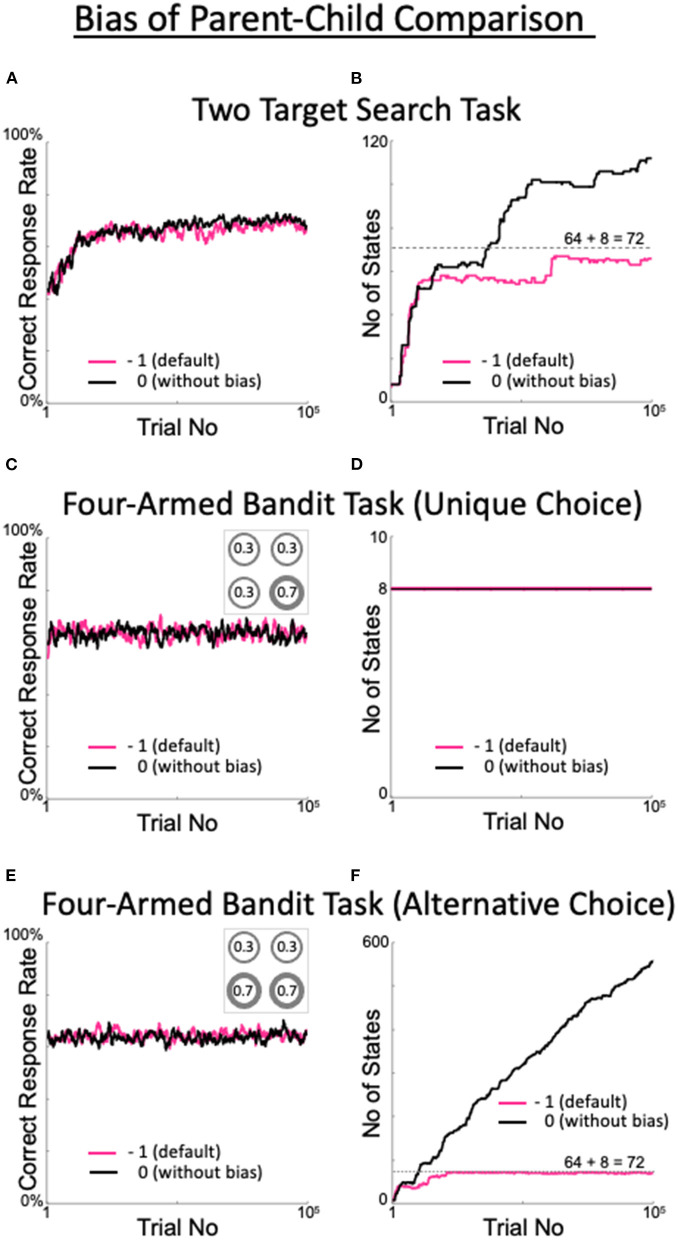
Effects of the presence or absence of the parent–child comparison bias. **(A,B)** Percentage of correct answers **(A)**, and changes in the number of states **(B)** in the two-target search task. **(C,D)** The same plots for the four-armed bandit task. The number in each circle in the inset of C represents the reward probability for each target. **(E,F)** The same plots for the alternative version of the four-armed bandit task. This version includes the two targets with the highest reward probabilities, as shown in the inset in **(E)**.

In the calculations of the present model, a relatively strict criterion was set for state expansion. That is, we used a bias when comparing the D_KLD values of the parent and child states (Equation 9). If the degree of decision uniqueness of the child state was not significantly improved over that of the parent state, the child state was pruned. However, when learning the two-target search task, the percentage of correct responses in the absence of a bias (black line in Figure 9A) was comparable to the high percentage in the presence of a bias (red line in Figure 9A). As expected, in the absence of a bias, the number of generated states increased, although not constantly (black line in Figure 9B). These results indicate that the default values of the four above-discussed meta-parameters have high validity.

To check the generality and applicability of the default values of the meta-parameters, we ran a four-armed bandit task and examined model performance. In the bandit task, four targets used in the two-target search task were assigned distinct reward probabilities (Figure 9C inset). When the optimal target selection was uniquely determined, i.e., when there was a single target with the largest reward probability (Figure 9C inset), the model quickly learned the behavior that yielded the largest correct response rate, regardless of the presence or absence of a bias (Figure 9C). In this case, the number of states remained minimal (determined as 8, depending on the model configuration; Figure 9D). The model learned to select only the target with the maximum reward probability and thus did not expand the number of states. The performance indicates that our model, and the values of the meta-parameters used therein, are generally and broadly applicable.

When there were multiple targets with the maximum reward probability (in this case, two: Figure 9E inset), the correct response rate was high (70%) in the presence or absence of a bias (Figure 9E), while the change in the number of states differed greatly between cases with and without a bias (Figure 9F). In the presence of a bias, the number of states did not exceed 72, and fewer than three trials were referred to. This result is generally plausible. In the bandit task, where there is no history of reward probabilities, referencing up to two trials does not improve the uniqueness of the action decision over the parent state, where only the last trial is referenced. After the eight states referring to the previous trial (which are not pruned according to our model) are saturated with experience, the states referring to the previous two trials are repeatedly generated and pruned. The behavior of the model without a bias is also reasonable. In the absence of a bias, child states are not pruned if their decision uniqueness is nearly equal to but slightly less than that of their parent state. Therefore, the model refers back to increasingly larger numbers of trials in search of a state that can deterministically select its action. The question of whether it is better to have bias is addressed in the Discussion section.

### Adaptability to an Unexpected Behavioral Task

**Figure 10:**
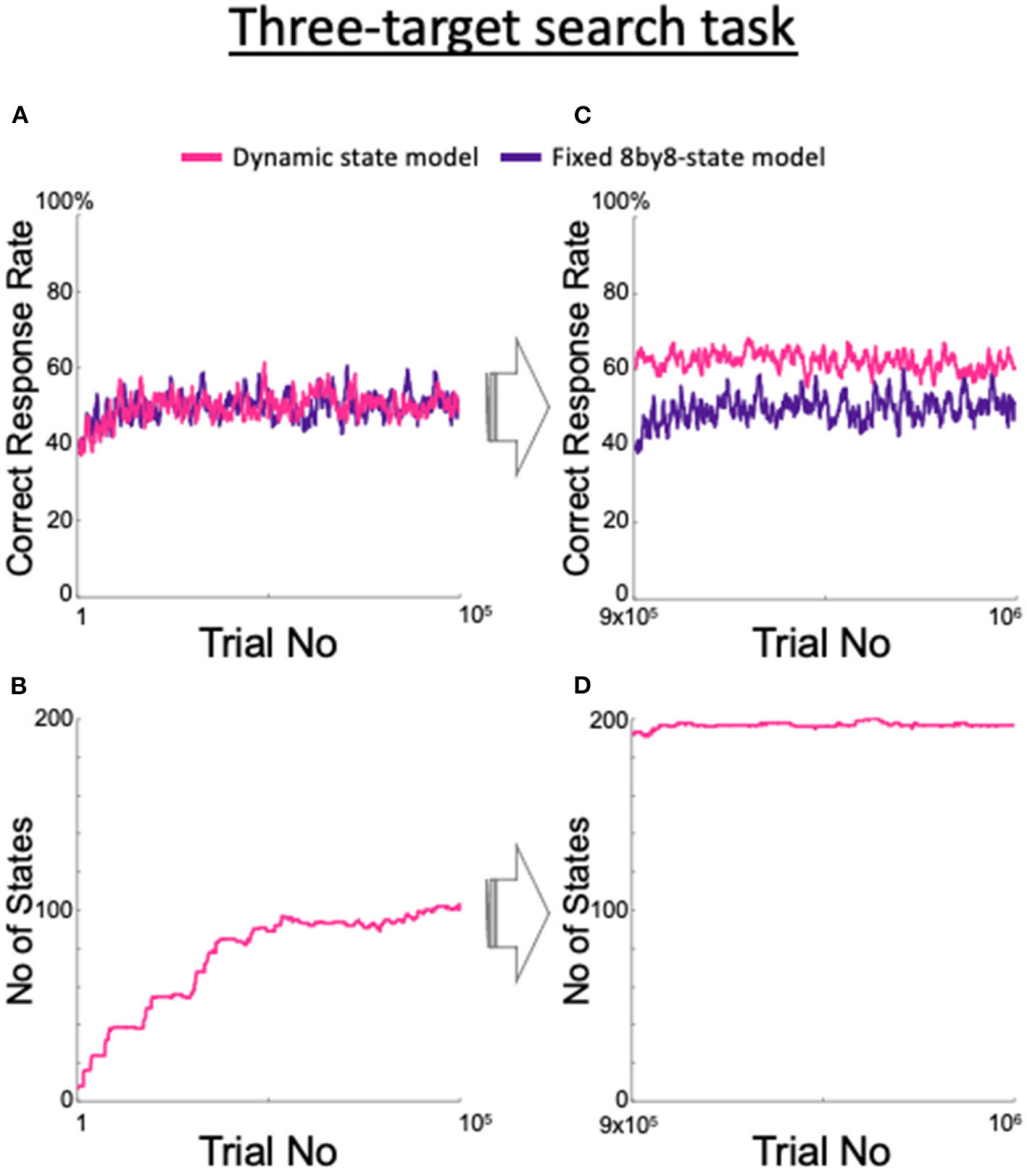
Changes in the proposed model as it learned a three-target search task and comparison with the fixed 8by8-state model. **(A)** Correct response rate for the first 10^5^ trials. **(B)** Increase in the corresponding number of the states. **(C)** Correct response rate for the 9 × 10^5^th trial to the 10^6^th trial. **(D)** Corresponding number of states.

We examined the adaptability of the model to unexpected situations and found that our proposed dynamic state model adapted to unexpected changes in the task requirements. We trained the fixed 8by8-state model, which is an ideal model for the two-target search task, and also the dynamic state model to perform a three-target search task that has not been attempted in monkeys (Figure 10). In this task, three of the four spots were the correct targets in a clockwise or counterclockwise order; the valid three-spot set was switched after seven consecutive correct trials, as in the two-target search task. Although there was no significant difference between the two models until after 100,000 trials (Figure 10A), Figure 10B shows that the dynamic state model steadily increased its number of states. As the number of trials increased, the fixed 8by8-state model showed no increase in the correct rate, while the dynamic state model demonstrated a steady increase (Figure 10C) due to the increase in the number of states (Figure 10D). Although the rate of increase in the number of states slowed compared with the rate during the first 100,000 trials, the increase continued after the millionth trial (data not shown).

**Figure 11:**
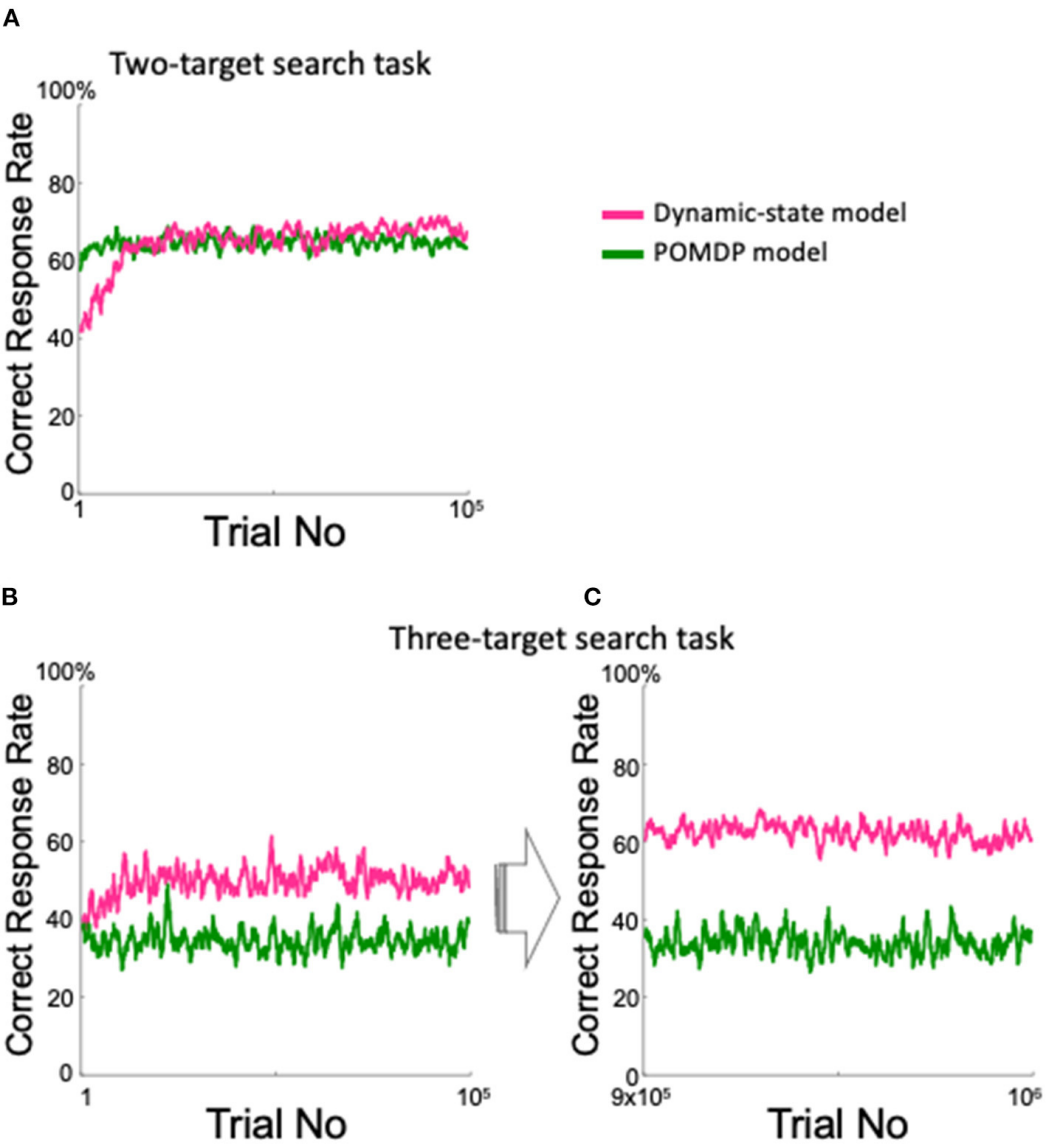
Comparison between the proposed model and the POMDP model. **(A)** Correct response rate in the two-target search task. **(B,C)** Correct response rates in the three-target search task for the first 10^5^ trials **(B)** and for the 9 × 10^5^th to the 10^6^th trials **(C)**.

We also created a simple POMDP model that was intended to perform well in the two-target search task. The model inferred which pair was currently the valid pair, although it only referred to the previous trial. As expected, the model performed in a manner comparable with the dynamic state model for the two-target search task (Figure 11A). However, in the three-target search task, the POMDP model showed lower performance than did the dynamic state model throughout the first 100,000 trials (Figure 11B); unlike the dynamic state model, the POMDP model did not show any improvement after nearly one million training trials (Figure 11C).

**Figure 12:**
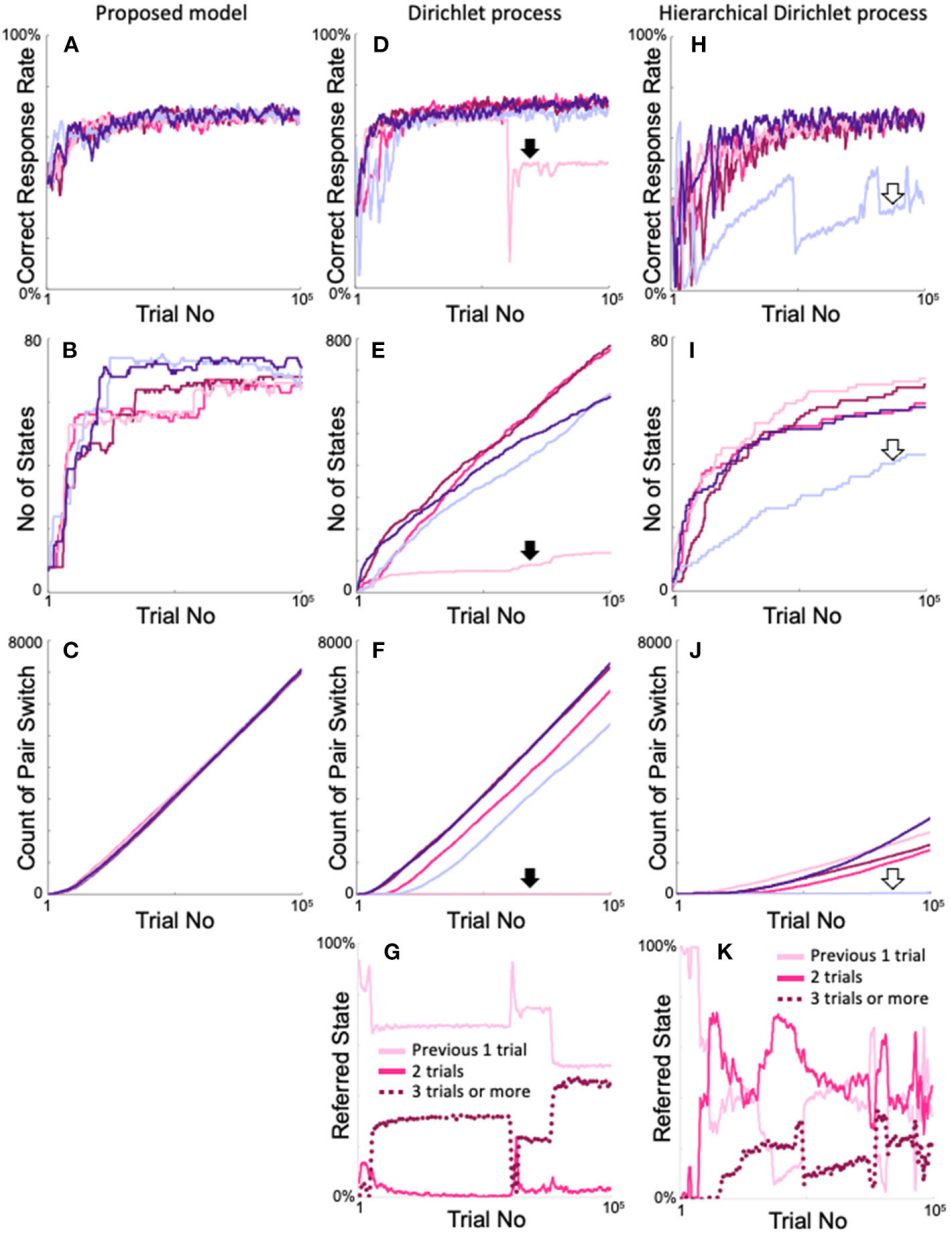
Comparison of the proposed model and the iHMMs in terms of the reproducibility of two-target search task learning. **(A–C)** Time courses of the correct response rate **(A)**, increase in number of states **(B)**, and increase in the cumulative number of target pair-switches **(C)** exhibited by the proposed model. **(D–F)** Identical plots of the Dirichlet process version of the iHMM (see Supplementary Figure 2C). **(G)** Changes in states with each action selection in the calculation example indicated by the filled arrows in **(D–F)**. **(H–J)** Identical plots of the hierarchical Dirichlet process version of the iHMM (see Supplementary Figure 2D). **(K)** Plot identical to G for the calculation example indicated by the blank arrows in **(H–J)**. The same color in the simulations of each model denotes the same calculation.

Furthermore, we compared our models with other iHMMs that also generate arbitrary numbers of states without prior knowledge of the two-target search task (Supplementary Figure 2). Figure 12 shows the time dependences of the correct response rate (Figures 12A,D,H), number of states (Figures 12B,E,I), and cumulative pair switching counts (Figures 12C,F,J) when the proposed model, Dirichlet version of the iHMM, and hierarchical Dirichlet version of the iHMM were trained five times each on the two-target search task. The proposed model showed stable and good performance in the two-target search task. The change in correct response rate was almost ideal (over approximately 20,000 trials) in all five calculations (Figure 12A). Correspondingly, the number of states increased rapidly but did not exceed 8 + 8^2^ = 72, which corresponds to the case of referring up to two trials (Figure 12B). The model exhibited smooth pair switching, and after 100,000 trials, achieved over 7,000 pair switches with good reproducibility (Figure 12C).

The Dirichlet model also exhibited ideal trends in correct response rates (Figure 12D). However, in some cases, performance declined rapidly during the learning process, once every few calculations (pale red area in Figure 12D, indicated by a bold arrow). In many cases, the number of states continuously increased. Consequently, 600–800 states that are unnecessary to perform the two-target search task were generated (Figure 12E). In contrast, in some cases, the model failed to increase the number of states (shown by the pale red area in Figure 12E, also indicated by a bold arrow). This calculation example achieved very little pair switching (Figure 12F pale red line with a filled arrow). In the other examples, the model steadily increased the cumulative count of pair switches, but did not show the same reproducibility as the proposed method (Figure 12F). In the example calculation of poor performance shown in Figures 12D–F as pale red lines, sharp variation existed in the number of reference trials (Figure 12G).

The Dirichlet model was compared with the proposed model, which used a neutral initial *Q*-value of 0.5 (see Figure 3B) when a new state was generated. For fairness of comparison, a neutral distribution was used in the Dirichlet model (see Method and Supplementary Figure 2C) as the initial value when a new state was generated. In contrast, the hierarchical Dirichlet models, which is more common than the Dirichlet model, inherit the parent distribution when a new state is generated, as schematized in Supplementary Figure 2D. Figures 12H–K show the results of the hierarchical Dirichlet model. The model exhibited a generally slower increase in the correct response rate than the above two models, but the rate became high near 100,000 trials (Figure 12H). We also encountered a calculation case where the correct rate deteriorated rapidly (Figure 12H, pale blue line indicated by a blank arrow). As expected, the increase in number of states was, in general, much smaller than that of the Dirichlet model (Figure 12I), because new states inherit the experienced distribution of their parents; consequently, the probability of generating a new state was low. Also, as in the Dirichlet model, the increase in the number of states was significantly smaller in the calculations that showed poor performance than in the other calculations (pale blue line in Figure 12I indicated by open arrows). Overall, the cumulative count of pair switches decreased significantly (Figure 12J), whereas the example defective calculations also exhibited almost no pair switches (pale blue line in Figure 12J). The example denoted by the pale blue line of Figure 12I had fewer than 72 states, but this does not mean that only the last two trials were referred to. Figure 12K shows the time evolution of the referred state exhibited by the defective calculation example denoted by the pale blue lines in Figures 12H–J. The model frequently took states that refer to more than two trials (dotted line in Figure 12K), which implies that a large number of states referred to more than two trials and an insufficient number of states referred to the optimal two trials on the two-target search task. Moreover, Figure 12K shows that the states referring to one, two, and three or more trials were rapidly switched during training, which differs from the stable behavior of the proposed model (Figure 4C). In conclusion, the Dirichlet and hierarchical Dirichlet models show not bad but unstable performance, in contrast to our proposed model.

### Exploration–Exploitation Trade-Off

The balance or trade-off between exploration and exploitation is recognized as a major challenge in reinforcement learning (Sutton and Barto, 1998). To maximize the total reward amount when executing a task, agents should neither rely very rigidly on their prior successful experiences (exploitation) nor select actions in an excessively arbitrary manner (exploration). The two-target search task includes exploration and exploitation phases. Therefore, our task is suitable for studying the exploration–exploitation trade-off problem by examining the relationship between the amount of learning in the model and the perseverative tendency with respect to the previously valid pair.

**Figure 13:**
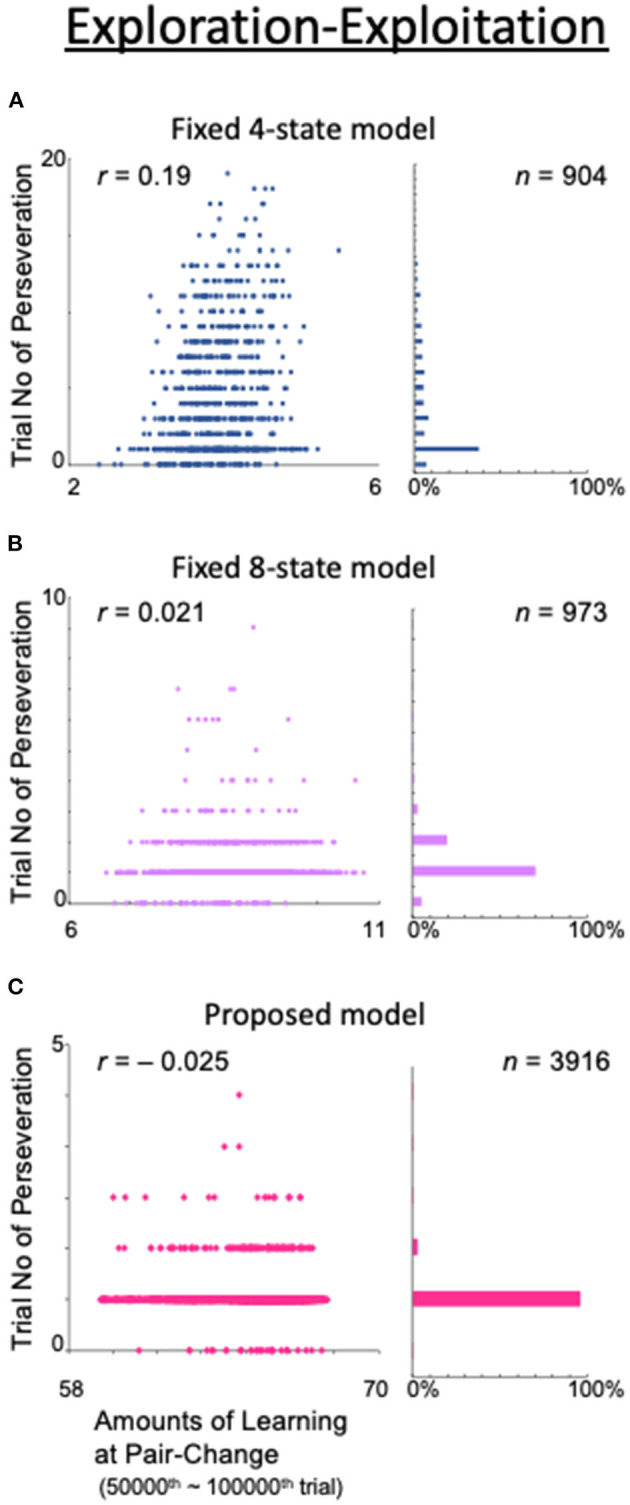
Analysis of the exploration–exploitation trade-off problem. (Left column) Amount of learning at the end of the last exploitation phase (abscissa) and the number of consecutive trials during which the model exhibited an action that persisted from the previous valid pair (ordinate). (Right column) Histograms of trials with perseveration as a percentage of the total number of trials. **(A)** Fixed 4-state model. **(B)** Fixed 8-state model. **(C)** Proposed model.

To examine this trade-off problem, we obtained the correlation between the total amount of learning at the time of a valid-pair switch and the number of consecutive trials for which the action adhered to the most recent valid pair (Figure 13). The correlations were calculated from the 50,000th to the 100,000th trial, when the correct response rate was considered sufficiently stable based on Figure 4A. The initial value of *Q* for each state was set to 0.5, so the total learning was defined as the sum of the absolute values of the differences from 0.5 within the *Q* table. For example, if the model has four states, {0.1, 0.7, 0.2, 0.6}{0.1, 0.7, 0.2, 0.6}{0.1, 0.7, 0.2, 0.6}{0.1, 0.7, 0.2, 0.6}, the total amount of learning is (|0.1 – 0.5| + |0.7 – 0.5| + |0.2 – 0.5| + |0.6 – 0.5|) × 4 = 4. In the fixed 4-state model, the state is based only on what was seen in the previous trial, i.e., the result is not considered (Figure 13A). This model naturally had a low rate of correct answers (data not shown) and, as a result, the number of pair-switching trials was as low as 904 in this calculation example. The number of trials with perseveration was also high (up to 20), and a stronger correlation between the total amount of learning and this number (*r* = 0.19) was observed than in the models shown in Figures 13B,C. This indicates that, in the fixed 4-state model, a high total amount of learning was associated with more difficulty in switching to exploratory behavior. In other words, there was a trade-off between exploration and exploitation.

However, in the fixed 8-state model (Figure 4A), in which the choice of action was based not only on the action of the previous trial but also on the result thereof, as well as the number of trials with perseveration, the correlation between the total amount of learning and this number was also greatly reduced (*r* = 0.0021; Figure 13B). However, the number of valid-pair switches did not significantly increase (*n* = 973), because this model required a large number of trials to obtain a deterministic behavioral decision.

The dynamic state also allowed the model to avoid the exploration–exploitation trade-off. In our proposed model, although the total amount of learning increased with the number of states, the number of trials with perseveration decreased further, and the number of valid-pair switches increased dramatically (*n* = 3,916). Concerning the correlation between the total amount of learning and the number of trials with perseveration, a small, or even negative, value was found (*r* = −0.025; Figure 13C). These results indicate that by including the results of the action in the state, the models learned how to take an action when they made a mistake—they learned how to explore.

## Discussion

In this study, we developed a reinforcement learning model with a dynamic state space and tested its ability to execute a two-target search task in which the exploration and exploitation phases alternated. To obtain a high score in this task, it is necessary to select an action according to the actions and reward outcomes of the two previous trials. The proposed model was able to dynamically and reproducibly expand and contract the state space based on two explicit criteria for the appropriateness of state expansion: experience saturation (ζ) and a preference for deterministic action or decision uniqueness (η). Thus, it demonstrated high performance, comparable with the performance of an ideal model with a fixed state space specific to the task, although it did not have a state space suitable for the task in advance. In addition, regardless of a behavioral task structure that was not assumed a priori, the proposed model exhibited an improvement in performance that could not be achieved with the fixed state model described above. Furthermore, by learning how to explore during the exploration phase, the proposed model did not exhibit a trade-off between exploration and exploitation.

### Validity of the Proposed Model

As shown in the behavioral analysis in Kawaguchi et al. (2015), monkeys were smart enough to learn to switch their behavior reflecting their own previous actions and their results (Shima and Tanji, 1998), rather than to learn by trial and error which spots to look at each time the correct target is changed. Therefore, it was necessary for the foundation of the proposed model, or the main models for comparison to highlight the characteristics of the proposed model (specifically, the Fixed 8by8-state model and the Fixed 8-state model), to have the actions taken by the subject and their results as the states. It may not be the usual manner in the field of reinforcement learning to define the state space the same as the action space. The state space usually corresponds to perceptual information, which in the case of Figure 2A would be the single state of presentation of the four white spots. However, it is obvious that the performance of a model using such a single state that learns to shift its gaze by trial and error is far less than that of monkeys, and discussions based on such a model are not fruitful. Furthermore, even if the action space into the state space were to be incorporated, the majority of RL researchers would use four states, that is, states based on the definition of “seeing one light spot among four identical stimuli” in the case of the two-target search task, rather than using eight states that the main models in this study based on. In fact, because we understand this point, we also showed calculations in Figure 13A for the model including the fixed four states. However, there is not much to be gained by using that model as the main comparison for the proposed model, for the same reason as mentioned above.

The dynamic state model performed as expected. Analysis of the state space dynamics (shown in Figures 4B,C) revealed that the model appropriately handles state space, which it readily expands or contracts. The performance in the multi-armed bandit task (Figures 9C–F) also indicates that the model did not extend states if their decision uniqueness is not better than that of their parent states. The dynamic state model is also robust. The model meta-parameters (α, β, ζ, and η) have a sufficiently wide range to enable the model to perform well (Figures 5–8). Even without the bias of parent–child comparison, the model still produces a high correct rate (Figure 9A) and a slightly high, but limited, number of states (Figure 9B) in the two-target search task, while it shows high correct rates (Figures 9C,E) and the expected numbers of states (Figures 9D,F) in the multi-armed bandit tasks.

The proposed model has an intrinsically greater ability to adapt to an indefinite environment, compared with the POMDP model requiring prior assumption at least for the probability space. Indeed, in the two-target search task, the POMDP model showed a high correct rate by estimating the current valid pair given as prior knowledge, although it only referred to the previous trial (Figure 11A). However, in the three-target search task, where prior knowledge of the valid pair was irrelevant, our dynamic state model performed much better than did the POMDP model (Figures 11B,C).

The iHMMs illustrated in Figure 12 are probably the closest to the proposed model, as they do not require the assumption of prior knowledge of the environment. These models follow the Dirichlet process and dynamically expand the state according to the history of each action taken in the state. However, unlike the proposed model, there is no explicit criterion for determining the appropriateness of state expansion; therefore, the state is not always easily expanded when it should be, and may be easily expanded when it should not be. As a result, the number of recent trials referred to in the action decision is unstable (Figures 12G,K), resulting in less reproducibility of the correct response rates (Figures 12D,H), a higher number of states (Figure 12E), and a low reproducibility in the cumulative number of pair-switches (Figures 12F,J) in the two-target search task compared with the proposed model. Decision uniqueness provides the purpose of state expansion, and experience saturation determines the timing of state expansion. These two criteria regulate the appropriateness of state expansion, resulting in flexible and stable model performance.

Decision uniqueness is related to an orientation toward causal determination or causal attribution (Heider, 1958; Kelley, 1967, 1973). To our knowledge, no published models consider experience saturation and a preference for deterministic action or decision uniqueness in reinforcement learning, although it is quite reasonable to assume them. For example, doctors clearly require sufficient study and experience to be able to properly diagnose patients; doctors would never roll the dice, despite encountering a patient who is difficult to diagnose. Each doctor would consult his or her colleagues and study relevant literature to make a specific, unique decision. The validity of decision uniqueness is supported by behavioral studies: this orientation has also been found in children (Schulz and Sommerville, 2006); Rodents recruit contextual information from the environment to uniquely determine the meaning of ambiguous CS (Fanselow, 1990). Furthermore, experience saturation is related to boredom, which is the counterpart to curiosity. In non-human primates, extensive experience with a task can lead to boredom (Harlow, 1950), as often observed by researchers who train monkeys to execute behavioral tasks. While it may be undesirable for artificial intelligence to exhibit “boredom,” this phenomenon is very common in humans. The drive toward state–space expansion based on these two criteria is an expression of curiosity; it reflects a tendency to deepen one's understanding of the environment.

### Implications of the Model's Behavior

Figure 4B shows a step-like increase in the number of states at approximately 60,000 trials. This corresponds to an increase in the diagonal gaze rate and a decrease in other gaze rates in the second trial of the exploration period (Figures 4D,E). However, these changes were slight, as were changes in the correct response rate (Figure 4A). The contrast between this small change and the rapid increase in the number of states is intriguing. This contrast may indicate that learning is not solely determined by environmental factors through correct and incorrect answers; it also reflects a process of refinement and maturation within the model. This observation may mean that, for example, each professional athlete appears to have a much higher level of knowledge and experience of the game, compared with amateurs; however, behind the slight differences in skill that determine who wins and loses lie large differences between players in terms of knowledge and experience.

The behavior of the model in response to outlier values of the meta-parameters is also important to note (Figures 5–8). When the criterion for experience saturation ζ is too low, the model tries to obtain more information from the existing state and thus does not expand the state space (dark purple line in Figure 5B). Conversely, when ζ was too high, the model easily gained what it considered sufficient information, the number of states generated was somewhat higher than the minimal required number (i.e., 72), though there was no uncontrollable increase in it (light purple line in Figure 5B). These findings indicate that neither insatiable learners nor learners who become too easily bored are ideal.

Somewhat similarly, if η was too low, even a *Q*-table with a sufficient preference for a single action was not regarded as deterministic and the state space was over-expanded to refer to the last three trials (yellow line in Figure 6B), though the correct rate itself was almost ideal. By contrast, if η was too high, the unique action selection was compromised, resulting in only eight states being retained, and thus referring to only the previous trial (brown line in Figure 6B). These observations may indicate that if learners try to decide things in an excessively strict manner, they may become mired in the tiny details of the situation; if learners are excessively irresponsible, they will not achieve a correct understanding of the environment.

The behavior of the model with outliers in the inverse temperature β of the softmax function can be understood in a manner similar to the case of η (Figure 8). If β was insufficient, the model chose actions randomly (i.e., in an irresponsible manner), which did not improve its understanding of the environment. Conversely, if β was excessive, the model persisted in certain actions, which limited the speed of its environmental understanding. Notably, the situation differed for the learning rate α (Figure 7). In particular, when the learning rate was high, the model learned the new valid pair quickly after pair switching, which did not lead to a good understanding of the task structure. This suggests that quick learners do not understand things deeply.

The bias in the parent–child comparison with a value of −1 suppresses the explosion in the number of states. Indeed, in the absence of a bias, the number of states is limited but more than the minimum required number (72) in the two-target search task, although there is no difference in the correct rates. In this sense, a bias is beneficial to the two-target search task. Given the change in number of states in the alternative version of the four-armed bandit task (Figure 9F), we consider whether it is preferable to have a bias. In the presence of a bias, increasing the number of states referring to the last two trials does not improve decision uniqueness; therefore, those states are pruned. However, this cannot handle the case where an action can be uniquely determined only after the last three or more trials have been considered. In contrast, in the absence of a bias, state expansion does not stop while the state is comparable to its parent state in terms of decision uniqueness. Therefore, when the task is essentially stochastic, as in this bandit task, it is not possible to stop state expansion. An intermediate method between the two is needed, which should be addressed in the future.

### Relationship of the Proposed Model to Related Works

The hierarchical Dirichlet model, which is compared with the proposed model in Figures 12H–K, is useful for language recognition problems, such as word estimation in sentences and word segmentation in Chinese and Japanese (Mochihashi and Sumita, 2007; Mochihashi et al., 2009). This model shows unstable performance in the two-target search task compared with the proposed model, although it often exhibits good performance. However, the rapid expansion of the state in the hierarchical Dirichlet model seems to be useful in problems such as language recognition, where the number of samples must be small, unlike the two-target search task where tens of thousands of trials can be sampled. The two criteria for the appropriateness of state expansion used in the proposed model are somewhat strict; if similar but more relaxed criteria are incorporated into the iHMM for language recognition processing, the model performance may improve.

As a learning architecture using KLDs, the free-energy principle has recently attracted considerable attention (Friston, 2009, 2010; Friston et al., 2009). This principle infers hidden variables in the environment such that free energy is minimized; specifically, predictions are maximized while allowing learners to actively work on the environment. KLD is used to maximize predictions; therefore, the computation aims to make no better predictions. This corresponds to the calculation of experience saturation in our model. It also may include active perceptual behavior (e.g., moving the eyes) to maximize prediction, which is consistent with our own behavior. However, this method is similar to the POMDP method in that it includes estimation of uncertain states, and the possible states are provided as prior knowledge. Thus, we cannot conclude that this principle is inherently equipped with the ability to adapt to indefinite environments.

Our proposed model attempted to extract complex temporal structures in the environment by using dynamic state space, similar to the reconstruction of dynamical systems in the field of non-linear dynamics. In particular, embedding is regarded as a method for identifying the underlying dynamics from time series data (e.g., Takens, 1981; Sauer et al., 1991; Ikeguchi and Aihara, 1995). For example, a chaotic dynamical system requires at least three dimensions. To reconstruct the trajectory of the chaotic system from the time series, two time intercepts (two-dimensional reconstruction map) are insufficient; three time intercepts (three-dimensional reconstruction map) are necessary. By applying the proposed model, we may be able to build a model that can learn to automatically reconstruct the non-linear dynamical system behind the time series data, just as our model could learn the task structure behind the three-target search task.

### Future Directions

The model proposed in this study showed a higher correct response rate in the three-target search task than the fixed 8by8-state model, which is an ideal model for the two-target search task (Figure 10). However, the difference between the two models did not become clear until approximately 1 million trials had elapsed. It took about 3 months (~100,000 trials) for the monkeys to master the two-target search task, including understanding of the events in a single trial, and the three-target search task is clearly more difficult than this task. However, in our experience, the three-target search task is easier than the path-planning and shape manipulation tasks that we had used in our previous experiments, which required more than 10 months of training (Mushiake et al., 2001; Sakamoto et al., 2008, 2015, 2020a,b). The reason why the model took so long to learn the three-target search task was because the optimal model for the three-target search task had to make a decision based on a combination of three trials, i.e., as many as 8^3^ = 512 states. In other words, the model faced the curse-of-dimensionality problem, where convergence slows as the number of states increases. To overcome this, the model will need the ability to generalize or abstract its experiences, such as “target three of the four points in order.” In fact, monkeys have much greater generalization ability, compared with our model, so they are expected to be able to learn the difficult tasks mentioned above. This generalization ability may correspond to the abstract representation of sequential actions by neuronal activities in the prefrontal cortex of monkeys (Shima et al., 2007; Sakamoto et al., 2020a).

In this paper, we dealt with a model in which one trial corresponds to one time step, but in the two-target search task that the animals actually performed, one trial included a sequence of events (Kawaguchi et al., 2013, 2015). It is not easy to build a model that can learn that more realistic latter situation, because we are faced with the problem of how to deal with one previous state. That is, it is unclear what constitutes one previous computation time increment; it could be one previous task event or one complete previous trial.

Einstein described his skepticism about quantum mechanics as follows: “Der Alte würfelt nicht (the old man does not roll the dice).” This expression seems to imply his desire for a deterministic understanding of the principles of the universe. Currently, artificial intelligence (AI), including reinforcement learning, is developing rapidly, and humans are delegating various decisions to AI. However, we do not want AI to roll the dice when we entrust it with important decisions. Instead, we want AI to constantly deepen its knowledge and experience, and to make deterministic decisions. The model proposed in this study did not exhibit a trade-off between exploration and exploitation (Figure 13). We hope that this model can serve as one of the foundations of AI that constantly deepens knowledge and experience, thus permitting deterministic decisions in complex and difficult environments.

## Data Availability Statement

The raw data supporting the conclusions of this article will be made available by the authors, without undue reservation.

## Author Contributions

TK, HM, and KS designed research. TK, MY, HH, and KS performed research and analyzed data. KS wrote the first draft of the paper and edited the paper. All authors contributed to the article and approved the submitted version.

## Funding

This work was supported by JSPS KAKENHI Grant Number JP16H06276 (Platform of Advanced Animal Model Support), 17K07060, 20K07726 (Kiban C), MEXT KAKENHI Grant Number 15H05879 (Non-linear Neuro-oscillology), 26120703 (Prediction and Decision Making), 20H05478 (Hyper–Adaptability) and Japan Agency for Medical Research and Development (AMED) under Grant Number JP18dm0207051.

## Conflict of Interest

The authors declare that the research was conducted in the absence of any commercial or financial relationships that could be construed as a potential conflict of interest.

## Publisher's Note

All claims expressed in this article are solely those of the authors and do not necessarily represent those of their affiliated organizations, or those of the publisher, the editors and the reviewers. Any product that may be evaluated in this article, or claim that may be made by its manufacturer, is not guaranteed or endorsed by the publisher.

## Supplementary Material

Supplementary Figure 1 The event sequence of one trial of the two target search task. Note that the actions in one trial and the results to them are defined as one time step, and the learning including the task sequence was not in this paper.

Supplementary Figure 2 The infinite hidden Markov models (iHMMs) compared to our proposed model. **(A)** Overview of the iHMM scheme. While the iHMM is similar to the proposed model (Figure 1C), it lacks a process to evaluate the appropriateness of state expansion. **(B)** Schematic tree showing how iHMMs expand the states. They start with a base state referring to no previous trials; each tree node stage corresponds to the reference trial number. Each state is the combination of the actions taken and their reward outcomes, as in the proposed model. **(C)** Schematic of the Dirichlet process version of the iHMM. The model was implemented using the Chinese restaurant process. Each filled circle (table) represents a possible action. If the action is rewarded, a new guest (a person with a filled head) sits at the table for that action. If an empty table is chosen, a new state is generated. In this case, a new state starts with the initial condition, in which each filled table has an “intrinsic guest” (a person with an empty head). **(D)** Schematic for the hierarchical similar Dirichlet process version. The newly generated child state inherits the distribution of the parent state. The *P*s in **(C,D)** represent the probabilities that each action is taken.
