# Supplementary figures and images for: Reinforcement Learning Model With Dynamic State Space Tested on Target Search Tasks for Monkeys: Self-Determination of Previous States Based on Experience Saturation and Decision Uniqueness
Source: Front Comput Neurosci. 2022 Feb 4;15:784592. doi: 10.3389/fncom.2021.784592 (PMC8855153; doi:10.3389/fncom.2021.784592)

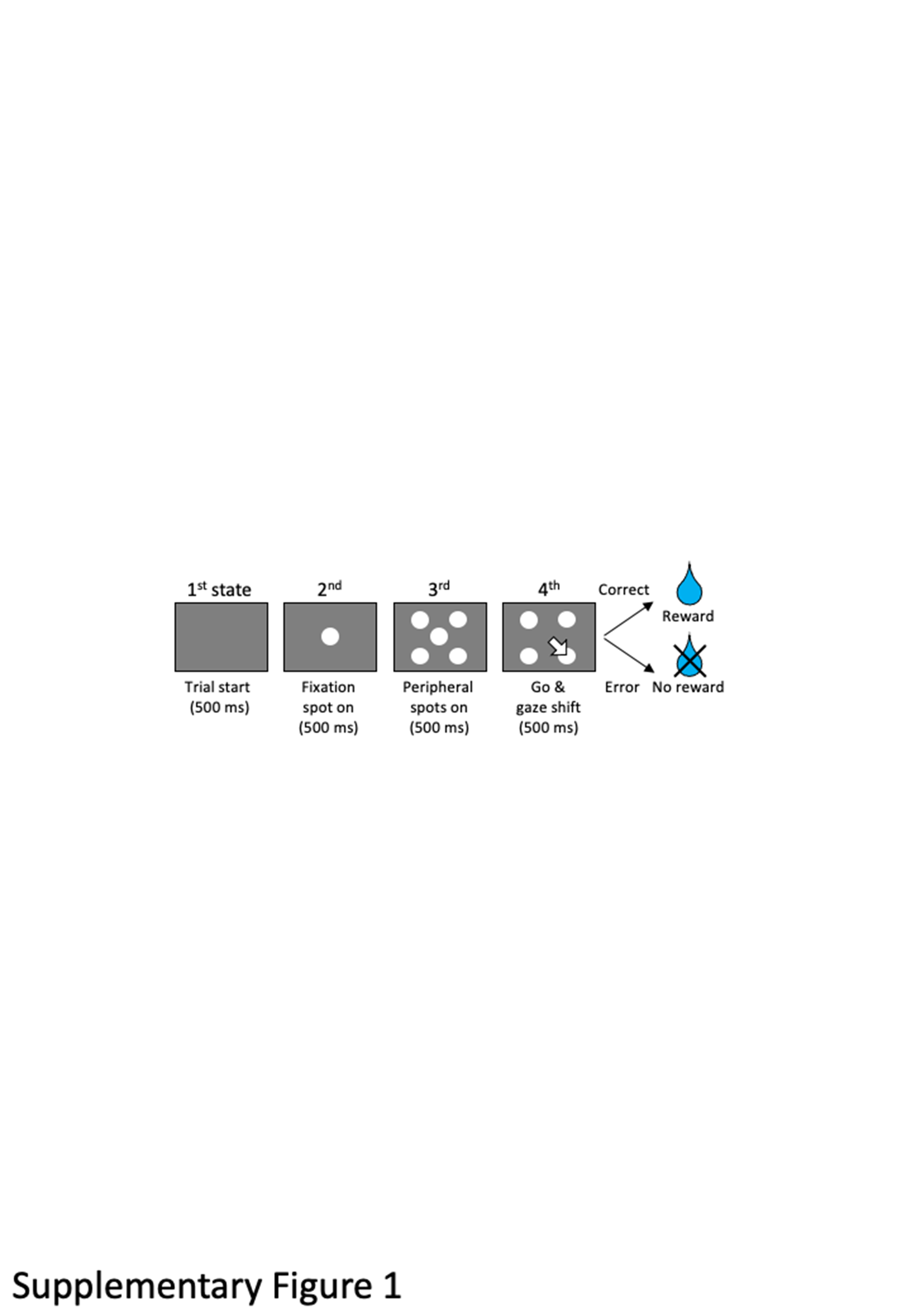

Supplement: Supplementary Figure 1 — The event sequence of one trial of the two target search task. Note that the actions in one trial and the results to them are defined as one time step, and the learning including the task sequence was not in this paper. [file Image_1.TIFF]

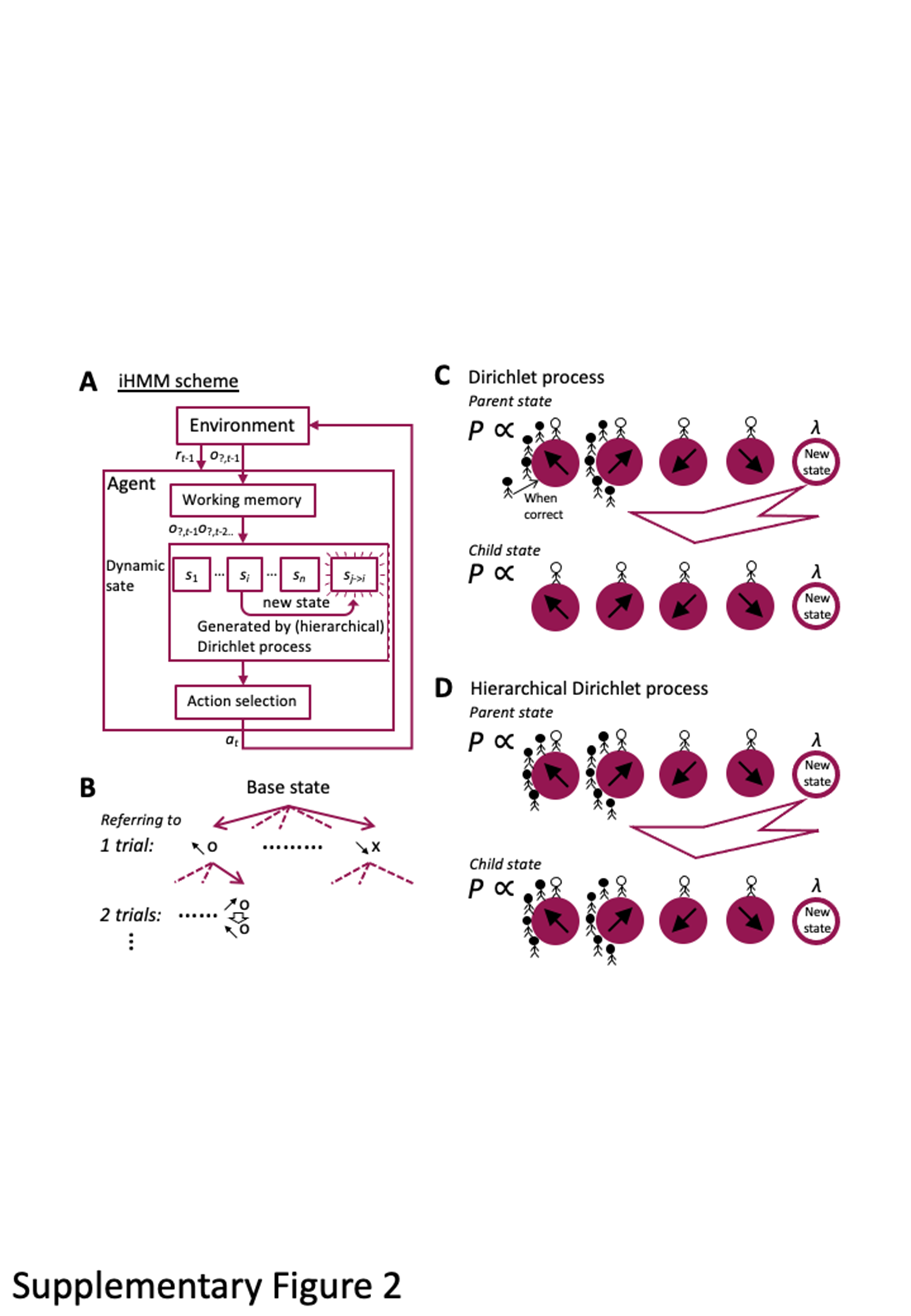

Supplement: Supplementary Figure 2 — The infinite hidden Markov models (iHMMs) compared to our proposed model. (A) Overview of the iHMM scheme. While the iHMM is similar to the proposed model (Figure 1C), it lacks a process to evaluate the appropriateness of state expansion. (B) Schematic tree showing how iHMMs expand the states. They start with a base state referring to no previous trials; each tree node stage corresponds to the reference trial number. Each state is the combination of the actions taken and their reward outcomes, as in the proposed model. (C) Schematic of the Dirichlet process version of the iHMM. The model was implemented using the Chinese restaurant process. Each filled circle (table) represents a possible action. If the action is rewarded, a new guest (a person with a filled head) sits at the table for that action. If an empty table is chosen, a new state is generated. In this case, a new state starts with the initial condition, in which each filled table has an “intrinsic guest” (a person with an empty head). (D) Schematic for the hierarchical similar Dirichlet process version. The newly generated child state inherits the distribution of the parent state. The Ps in (C,D) represent the probabilities that each action is taken. [file Image_2.TIFF]
